# Exploring the Impact of Biotic and Abiotic Surfaces on Protein Binding Modulation and Bacteria Attachment: Integrating Biological and Mathematical Approaches

**DOI:** 10.1021/acsnano.5c06573

**Published:** 2025-06-16

**Authors:** João Gabriel S. Souza, Martinna Bertolini, Jett Liu, Bruna Egumi Nagay, Rodrigo Martins, Raphael C. Costa, Jason Cory Brunson, Jamil Shibli, Luciene Cristina Figueiredo, Anna Dongari-Bagtzoglou, Magda Feres, Valentim Adelino Ricardo Barão, Batbileg Bor

**Affiliations:** 1 Dental Research Division, Universidade Universus Veritas Guarulhos, Guarulhos, São Paulo 07023-070, Brazil; 2 Department of Prosthodontics and Periodontology, Piracicaba Dental School, Universidade Estadual de Campinas (UNICAMP), Piracicaba, São Paulo 13414-903, Brazil; 3 Department of Periodontics and Preventive Dentistry, School of Dental Medicine, 6614 University of Pittsburgh, Pittsburgh, Pennsylvania 15260, United States; 4 Department of Microbiology, ADA Forsyth Institute, Cambridge, Massachusetts 02142, United States; 5 School of Dentistry, Alfenas Federal University, Alfenas, Minas Gerais 37130-001, Brazil; 6 Department of Pulmonary Systems Medicine, 3463 University of Florida, Gainesville, Florida 32610, United States; 7 Department of Oral Health and Diagnostic Sciences, University of Connecticut Health Center, Farmington, Connecticut 06030, United States; 8 Department of Oral Medicine, Infection, and Immunity, 124048 Harvard School of Dental Medicine, Boston, Massachusetts 02115, United States; 9 Faculdade Israelita de Ciências da Saúde Albert Einstein, Hospital Israelita Albert Einstein, São Paulo 05653-120, Brazil

**Keywords:** implant device, proteomic, microbiome, tooth, biofilm-related infections, surface adsorption

## Abstract

The oral environment is composed of a diverse array of proteins, and any substrate inserted into this habitat promptly becomes subjected to protein adsorption and bacterial colonization. However, the predictive and modulatory nature of implant surfaces coated with salivary pellicle proteomes in microbial adhesion has not been explored using high-throughput techniques. Thus, using human saliva for salivary pellicle adsorption and microbial accumulation, we compared adsorption and community formation on titanium (Ti) biomaterials (implant devices) and dental surfaces (enamel and dentine). The proteomic profile was evaluated by liquid chromatography coupled with tandem mass spectrometry, and the microbiome was assessed using 16S RNA sequencing. Linear discriminant analysis (LDA) and canonical correlation analysis (CCA) were used to quantify variation in analyte amounts and identify likely biomarkers. Substrates were analyzed regarding their physical, chemical, and topographical properties. Our results showed that the salivary pellicle proteomes on Ti exhibited differences in composition and protein intensities compared with dental surfaces. These differences in proteomes affected the biological processes at the level of microbiome accumulation. Geometric analysis showed greater similarity between Ti and enamel proteomes, while dentine differed markedly. Ti harbors a microbiome community that differs from that of dental surfaces. Canonical correlation analysis (CCA) pinpointed proteins that promoted or inhibited the adherence of specific microbes. Apolipoprotein E showed a strong negative correlation (>0.8) with . Higher levels of the protein on dental surfaces were associated with reduced microbial adhesion, whereas its absence on Ti surfaces facilitated increased bacterial adhesion. These findings provide valuable insights into the initial biological responses after the insertion of implanted devices, which can be leveraged by biomedical engineering to develop biomaterials with enhanced outcomes and prevent microbial accumulation.

## Graphical abstract

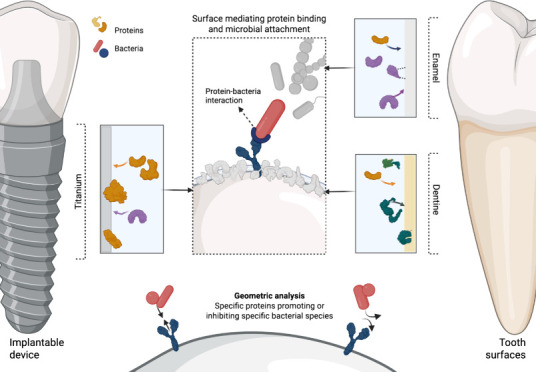

Protein adsorption is the first biological interaction between the human sites and solid biomaterial surfaces that form an interface with them. Adsorbed proteins affect subsequent cellular events, such as bacterial and host cell adhesion, and play a vital role in cell signaling, immune responses, and biomaterial-tissue bridging.^1–4^ Whole saliva, characterized by its richness in protein, exerts a critical influence on oral health and has recently emerged as an important source of biomarkers for various diseases and biological conditions.^5^ Upon insertion of any biomaterial into the oral environment, immediate protein adsorption occurs,^6,7^ primarily originating from saliva^2,3^ and contributing to the formation of a salivary pellicle. Interestingly, there is some overlap between saliva and blood plasma protein profiles.^8^ Whole saliva proteomes have been shown to be highly similar to salivary-pellicle proteomes in terms of composition, physicochemical properties, and molecular organization.^9^ Salivary pellicles forming on dental structures and abiotic surfaces, such as dental implant materials, act as receptors for microorganism adhesion and coaggregation.^7^ Consequently, the ability of a particular surface to adsorb specific proteins is directly correlated with surface-specific biological processes.^10^

The interaction between solid surfaces and proteins is affected by both the protein properties and the physical-chemical characteristics of the surface. Titanium (Ti) is an important biomaterial in the oral environment, considering its extensive use in manufacturing dental implant devices.^11^ Evidence shows that changes in topography and chemical composition of Ti-based implant devices^12–14^ or natural dental surfaces, including dentine and enamel (or hydroxyapatite),^15–20^ directly affect the proteomic profile of salivary pellicle. Earlier evidence has suggested lower and distinct protein adsorption on Ti surfaces compared to dental surfaces.^15,17,19^ Although some proteins have shown no significant difference,^16^ suggesting that despite heterogeneity in the overall protein profiles, there is an overlap in the core protein adsorption. However, these studies have certain drawbacks, such as their tendency to assess only a subset of selected proteins for binding, and they did not apply large-scale proteomic analysis, or did not consider whole human saliva.^15,17–19^ Therefore, to the best of our knowledge, a comprehensive evaluation of protein binding specificity to Ti, dentine, and enamel has not been explored when exposed to whole human saliva, with the use of high-throughput techniques.

Moreover, protein adsorption on surfaces constitutes the first step preceding microbial accumulation via adhesin and saliva protein (receptor) binding.^3,21^ Therefore, it becomes crucial to explore further the impact of proteomes in modulating and predicting microbiome signatures within the context of surface and biological system interactions. The human oral cavity provides a unique environment for microbial adhesion and polymicrobial aggregation, as it hosts 774 bacterial species.^22^ Salivary pellicles increase surface polarity and hydrophilicity and, consequently, increase bacterial attachment compared to noncoated surfaces.^23^ Specifically, the adhesins, well-recognized bacterial surface proteins, including the serine-rich repeat protein family, antigen I/II, and FomA, have the capacity to recognize and mediate the attachment to salivary constituents on the pellicle, thereby promoting bacterial adhesion.^1^ This specific interaction between salivary pellicle proteins and the initial colonizers promotes the selection and subsequent enrichment of certain microbial species to first attach to biomaterial substrates. Previous evidence has reported some differences related to initial microbial communities colonizing dental surfaces and implant devices immediately after their insertion.^24,25^ Importantly, while some proteins facilitate microbial adhesion, others may reduce bacterial physicochemical interactions, directly modulating biofilm formation.^26^ So far, no previous studies have explored how the salivary pellicle proteome composition can predict which initial colonizer will adhere and form a biofilm on Ti and dental surfaces (enamel and dentine).

In this study, we analyzed the proteomic composition, followed by the microbiome, of salivary pellicles on Ti, dentine, and enamel surfaces after exposure to whole human saliva. Our study was designed to rigorously elucidate how this proteomic profile influences further microbial adhesion and the initial formation of polymicrobial biofilms. By applying geometric analytical methods to biological experimental data, we aimed to predict the complex interplay between proteins adsorbed onto different surfaces and subsequent site-specific bacterial attachment to each coated substrate. Through this investigative approach, we sought to unravel the mechanistic role of surface characteristics in driving the dynamics of protein adsorption, thereby influencing the initial stages of microbial interaction and biofilm development. Our findings showed pivotal differences in terms of salivary pellicle proteomic composition and microbial accumulation patterns between implantable devices and dental surfaces. Geometric analysis revealed that specific proteins were strongly correlated with either promoting or inhibiting the growth of certain bacterial species, aligning with our biological data. These biomarkers can potentially guide the development of innovative biomaterial devices engineered to elicit enhanced biological responses and effectively control microbial infections.

## Results

### Physical and Chemical Properties of Biotic and Abiotic Substrates

We used biological and geometric statistical methods to explore how common substrates present in the oral environment (dental surfaces and Ti biomaterial) modulate protein adsorption from saliva and how this predicts microbial adhesion and accumulation. Enamel and dentine constitute the two primary components of dental surfaces, while Ti serves as the biomaterial used in dental implant manufacturing, the primary and main therapeutic solution for replacing missing teeth. Although dentine and enamel exhibit certain topographical similarities, confocal imaging revealed that dentine had greater surface roughness and area, primarily due to the presence of irregular regions associated with dentinal tubules (Figure 1A). These features were even more pronounced in Ti compared to enamel. In fact, AFM analysis showed a smooth and regular pattern for the enamel substrate, which is expected due to its mineral nature, and an irregular profile for dentine, with a notable presence of tubules in conical-shaped forms throughout the substrate (Figure 1B). The Ti substrate demonstrated a characteristically rough surface, with prominent peaks and valleys also revealed by AFM (Figure 1B). Therefore, the substrates presented distinct topographical patterns in terms of their physical properties. Although all substrates exhibited a hydrophilic profile, the Ti material showed significantly increased wettability compared to the other substrates, as evaluated by the water contact angle (Figure 1C). A slight trend toward higher roughness was observed for the Ti surface; however, it was not significant compared to enamel and dentine (Figure 1D).

**Figure 1:**
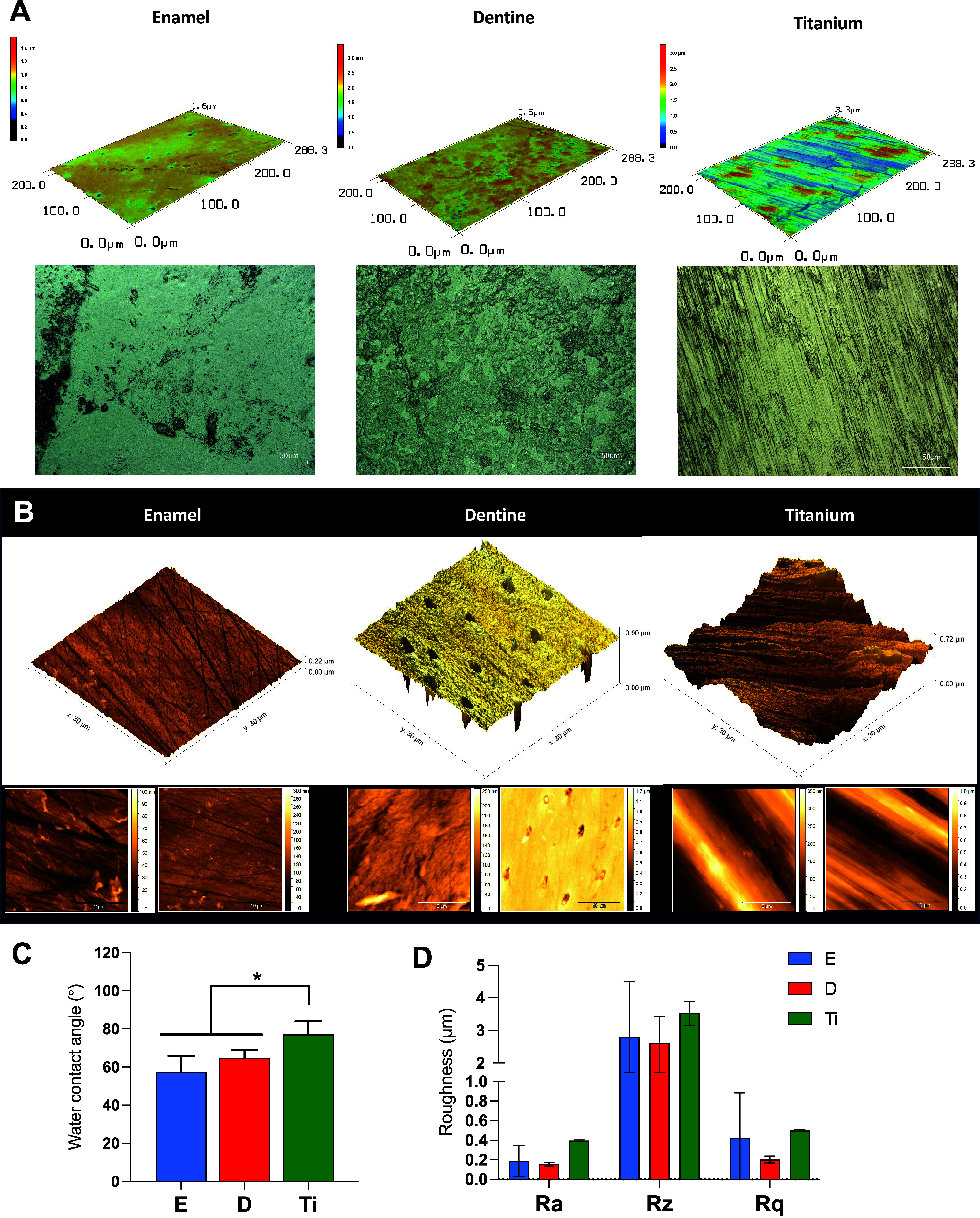
(A) Substrate topography was evaluated by a 3D laser scanning confocal microscope, and by (B) Atomic Force Microscopy (AFM) (scale 2 and 10 μm). (C) Wettability (°) measured by water contact angle. (D) Surface roughness (average ± standard deviation) by 3D laser scanning confocal microscope showing average roughness, *Ra*, root mean-square-average, *Rq*, and average maximum height of the profile, *Rz*. *p < 0.05. The error bars indicate standard deviations.

X-ray Photoelectron Spectroscopy (XPS) analysis showed that these substrates differ, not only in physical properties but also in chemical composition, especially when Ti is compared with dentine and enamel. The high-resolution XPS spectra of C 1s, O 1s, N 1s, Ca 2p, P 2p, and Ti 2p are shown in Figure 2. The C 1s spectra suggest the presence of organic constituents on enamel and dentin, and typical contamination peaks from environmental or cleaning processes for all groups. A main peak at 284.6 eV (enamel/dentin) and 285.5 eV (Ti) may correspond to C–C/C–H bonds from organic matrix components or surface hydrocarbons.^27^ Peaks at 288.3–288.6 eV, observed across all samples, may indicate C–O and O–C = O groups from adventitious carbon or carbonate species.^28^ Enamel also exhibited minor peaks at 280.1 and 283.6 eV, possibly related to low-binding energy carbon compounds. The N 1s peak at 399.9 eV suggests N–C bonds in proteins or peptides on the enamel/dentine surface. This signal is expected to correlate with the C 1s component at 286.2 and 286.4 eV, on enamel and dentine, respectively, which includes C–O–C and C–OH bonds.^29^ Regarding the O 1s spectra, for Ti surfaces, three distinct peaks were observed corresponding to O^2–^ ions at 530.4 eV, hydroxyl groups (OH^–^) at 532.3 eV, and adsorbed water (H_2_O) at 533.6 eV.^30^ In enamel and dentin, a peak at ∼ 532 eV was attributed to organic C = O bonds. Enamel also exhibited peaks at 528.9 eV and 531.0 eV, which may correspond to O^2–^ and phosphate groups (O–P), respectively, while a peak at 533.2 eV was related to adsorbed water.^29^ The Ca 2p peaks at ∼ 347 eV (Ca 2p_3_/_2_) and ∼ 351 eV (Ca 2p_1_/_2_) observed in enamel and dentine are characteristic of calcium in calcium phosphate compounds, such as hydroxyapatite.^31^ In contrast, P 2p peaks at ∼ 133.3 eV were detected only in enamel, consistent with phosphorus in phosphate groups (PO_4_^3–^), reflecting its higher phosphate-rich content.^31^ As expected, Ti 2p peaks were observed only on the Ti surface, with signals at 458.6 and 464.3 eV corresponding to Ti^4+^ in TiO_2_, and a lower-intensity peak at 453.5 eV indicating the presence of metallic Ti.^32^

**Figure 2:**
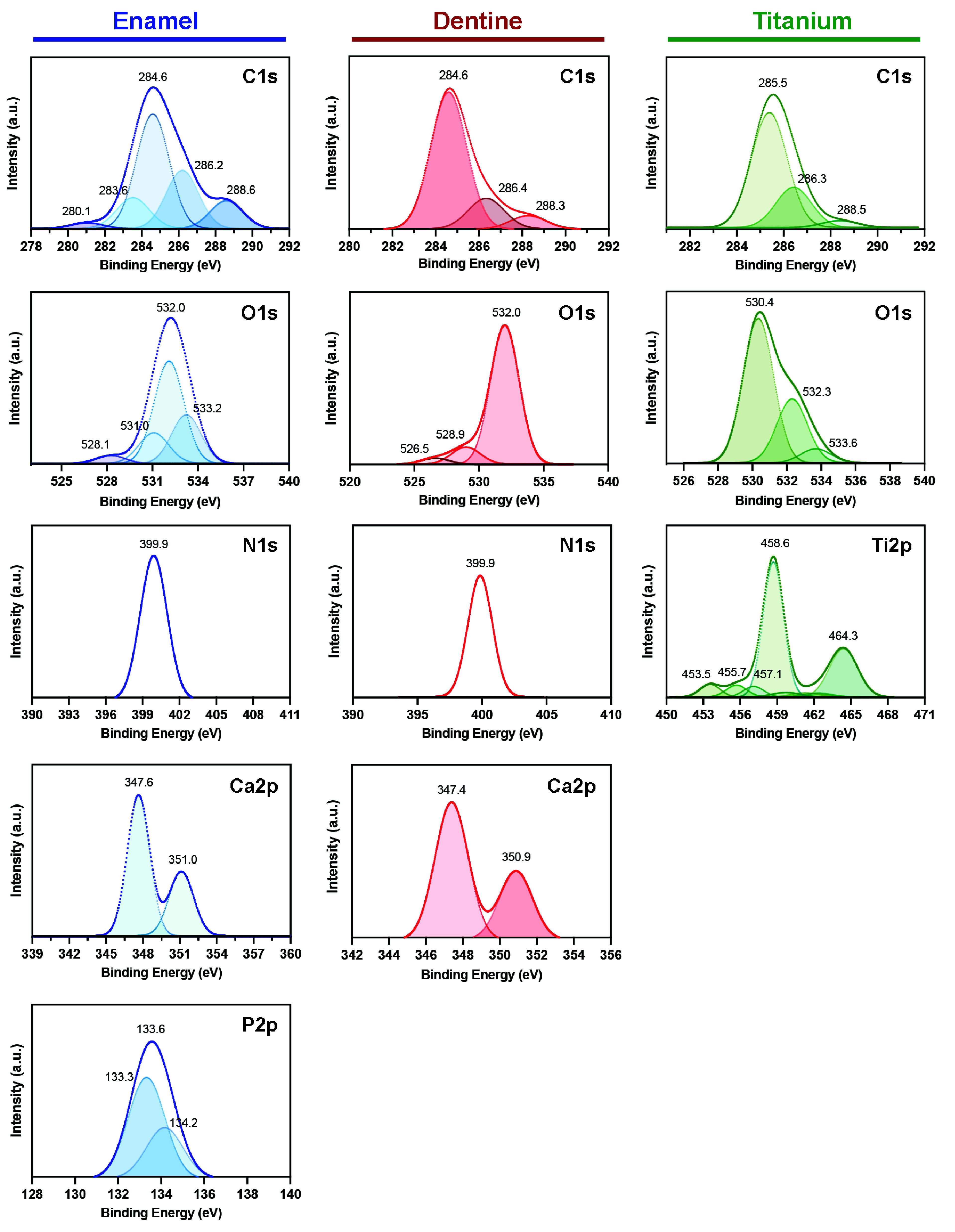
High-resolution X-ray photoelectron spectroscopy (XPS) spectra of the substrate surfaces, including C 1s, O 1s, N 1s, Ca 2p, P 2p, and Ti 2p regions. Each peak was deconvoluted to identify specific chemical states, and the corresponding binding energy values are indicated in the figure. Peak assignments were based on reference values from the literature and reflect the typical chemical environments expected for each material.

### Dental and Ti Surfaces Present Substantial Differences in the Composition and Intensity of Salivary Pellicle Proteomes

Considering the distinctions between dental surfaces and Ti, we evaluated how these properties contribute to the specificity of salivary protein adsorption. For this purpose, a salivary pellicle was formed on all substrates for 2 h, emulating the *in vivo* salivary pellicle maturation time. A total of 331 proteins were identified adsorbed onto the dentine, 249 on the enamel, and 297 on the Ti after exposure to the same pool of saliva (8 healthy patients) during salivary pellicle formation (Figure 3A). No statistical difference was identified among the substrates in terms of total average number of proteins identified by LFQ intensity (Figure 3B), as indicated by similar quantitative results across all substrates (Figure 3C). Although a high number of shared proteins was identified, the distinctive properties of each surface led to slight selectivity in terms of protein binding, resulting in some unique/exclusive proteins being adsorbed onto each substrate (Figure 3D). In pairwise comparisons, specifically comparing Ti with enamel and Ti with dentine, the Ti surface showed 58 and 15 unique proteins adsorbed, respectively (Figure 3D). The results indicated that even with exposure to a standardized whole saliva sample, the proteomic composition of the salivary pellicle was slightly affected by surface properties. Notably, exclusive proteins were identified for each substrate (Table S1 - Exclusive proteins identified in each surface). Enamel displayed a limited number (3) of exclusive proteins: keratin type II cytoskeletal 6B, E3 ubiquitin-protein ligase MIB2, and histone H1.5. Dentine showed the highest difference compared with the other substrates, presenting a total of 42 exclusive proteins adsorbed onto its surface.

**Figure 3:**
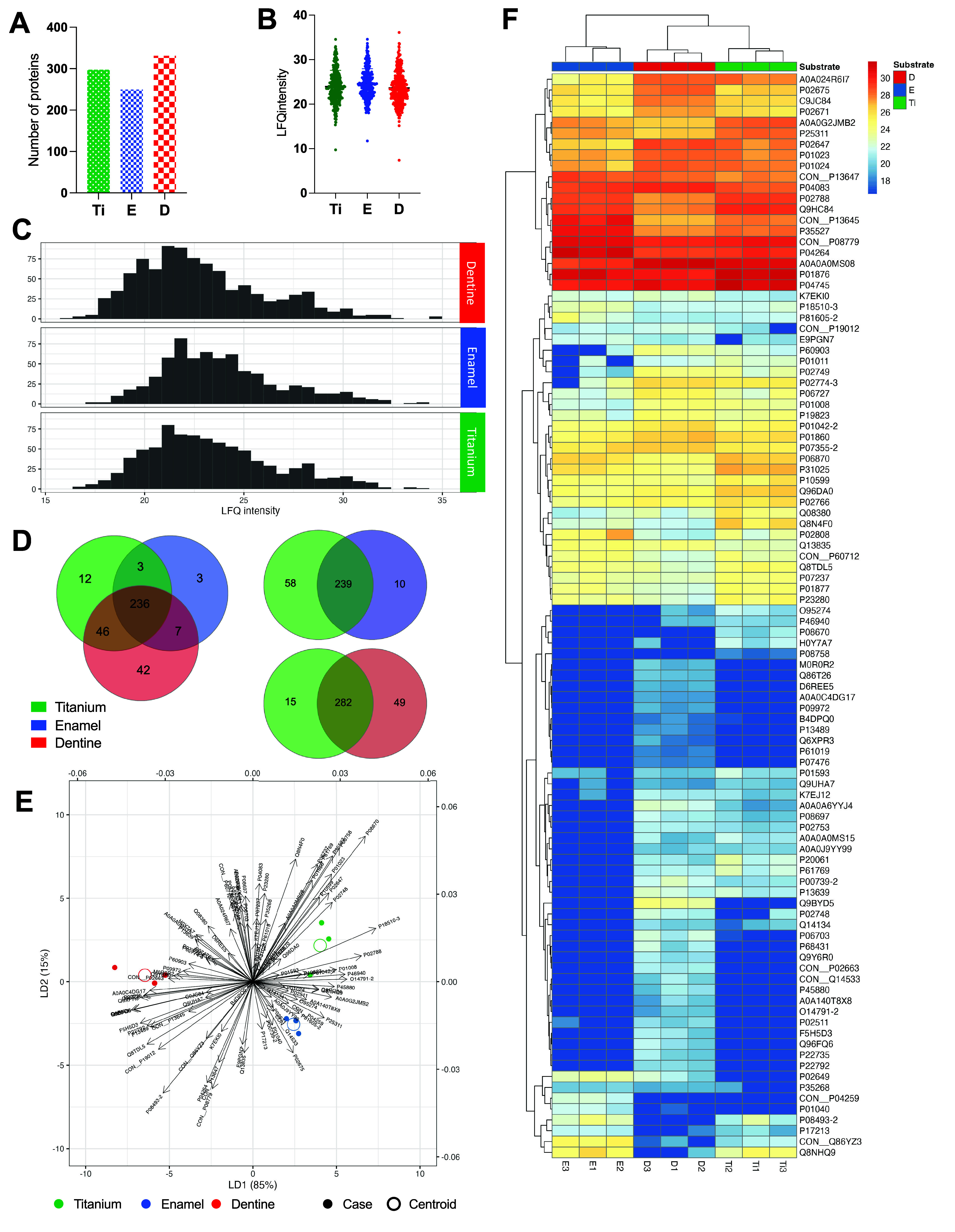
Proteomic profile of salivary pellicle adsorbed onto enamel, dentine, and Ti surfaces. (A) Total number of proteins identified for each substrate. After 2 h of protein adsorption using stimulated human saliva, the proteomic profile was evaluated by Liquid chromatography coupled with tandem mass spectrometry. (B) Average LFQ intensity of proteins identified for each substrate. (C) Histogram distribution of LFQ intensity of proteins identified for each substrate. (D) Venn diagrams comparing the substrates tested in terms of proteomic composition. Numbers indicated the number of proteins in each substrate and the shared number of proteins for circles overlapping. (E) Linear discriminant analysis (LDA) to quantify the discriminatory effect of each protein among the substrates. A two-dimensional biplot (LD1 and LD2) was generated to visualize the centroid and case scores together with the variable coefficients along both axes. The greater the alignment between the position of a substrate sample and the direction of a protein vector, the greater the differential intensity of that protein in that sample. (F) Clustered heatmap of shared proteins among the substrates with a statistical difference (*p* < 0.05, Kruskal–Wallis test) in terms of LFQ intensity. D - dentine; E – enamel; Ti – Titanium.

Linear discriminant analysis (LDA) was performed on the nine measurements of each of 110 proteins found to discriminate among the substrates by Kruskal–Wallis (KW) tests. The first discriminant axis, LD1, predominantly distinguished dentine protein profiles from Ti and, to a lesser extent, from enamel protein profiles, while LD2 primarily segregated Ti profiles from enamel profiles (Figure 3E). The biplot shows the three elements on the two linear discriminant axes: the profiles of protein intensities from each experiment (small disks - case), the mean profiles within the substrates (large circles - centroid), and the vectors of protein effects, the coordinates of which were their standardized discriminant coefficients along each axis (arrows). The greater the alignment between the position of a substrate sample (small disks and large circles) and the direction of a protein vector (arrows), the greater the differential intensity of that protein in that sample. The strongest discriminants along LD1 were P18510–3, P02649, Q96FQ6, Q9BYD5, and F5H5D3, while those along LD2 were P08670, P08758, P35527, Q8N4F0, and P61769. Interestingly, based on the LDA, there was greater similarity between Ti and enamel, with both substrates markedly differentiated from dentine (Figure 3E). A clustered heatmap showed the 110 shared proteins among the substrates with statistical difference (*p* < 0.05, KW test) in terms of LFQ intensity (Figure 3F). The row and column dendrograms encode complete-linkage hierarchical clusters based on Euclidean distances between measurements. These findings showed that Ti and dental surfaces were differentiated in terms of proteomic profile and intensity of proteins adsorbed from saliva. Contrary to expectations, based on the profile of the shared proteins, we concluded that dentine, rather than Ti, was the substrate exhibiting the greatest difference in protein adsorption profiles when compared with enamel.

To better understand the distinct protein signatures between natural and artificial substrates, we repeated the preceding analysis pipeline for these two groups, unifying dentine and enamel samples into one group and discriminating them from the Ti group. Interestingly, when comparing artificial (Ti) and natural (enamel and dentine) surfaces, remarkable differences in protein intensities were noted, influencing the composition of the salivary pellicle on these substrates (Figure S1 - Heatmap of shared proteins among the artificial and natural surfaces). A smaller number of proteins effectively discriminated between the natural and artificial substrates than those contributing to distinguishing among all three. In fact, there are important differences among the substrates in terms of protein intensities, considering all proteins identified (Figure S2 - LFQ intensity of all proteins identified).

Although there was a shift in the proteomic profile, mainly in terms of intensities, among the proteins adsorbed onto the substrates, the overall profile of molecular functions and biological processes mediated by these proteins was similar and consistent, but with some notable differences (Figure 4A). When considering the top 10 molecular functions and biological processes in terms of proportion (number of proteins), there was a slight variation among the substrates. Calcium ion binding, antigen binding, and structural molecular activity were the main molecular functions identified for Ti, enamel, and dentine, respectively. The main biological process mediated by proteins adsorbed on Ti was the immune response, while for enamel and dentine, it was keratinization. In the comparison between artificial (Ti) and natural (enamel and dentine) surfaces, LDA analysis identifies several molecular functions and biological processes with significantly different extents for the natural and artificial substrates, adopting a selection threshold of *p* < 0.05. Specifically, we identified 32 molecular functions and 47 biological processes that exhibited detectable differences in extent between those substrates. Figures 3B and C show the main molecular functions and biological processes that discriminate natural and artificial substrates. Negative coefficients along the first discriminant axis were associated with the artificial substrate, and positive coefficients were associated with the natural substrates (Figure 4B and C). Notably, the Ti substrate showed important molecular functions and biological processes related to the healing process, such as actin filament binding and inflammatory response, as expected for implant placement. Even among exclusive proteins on Ti, proteins related to immune response, such as Immunoglobulin kappa variable 2–30 and Coronin 1-A, were found. Therefore, Ti and dental surfaces were also differentiated in terms of molecular functions and biological processes mediated by proteins adsorbed from saliva, which may directly impact the subsequent biological events.

**Figure 4:**
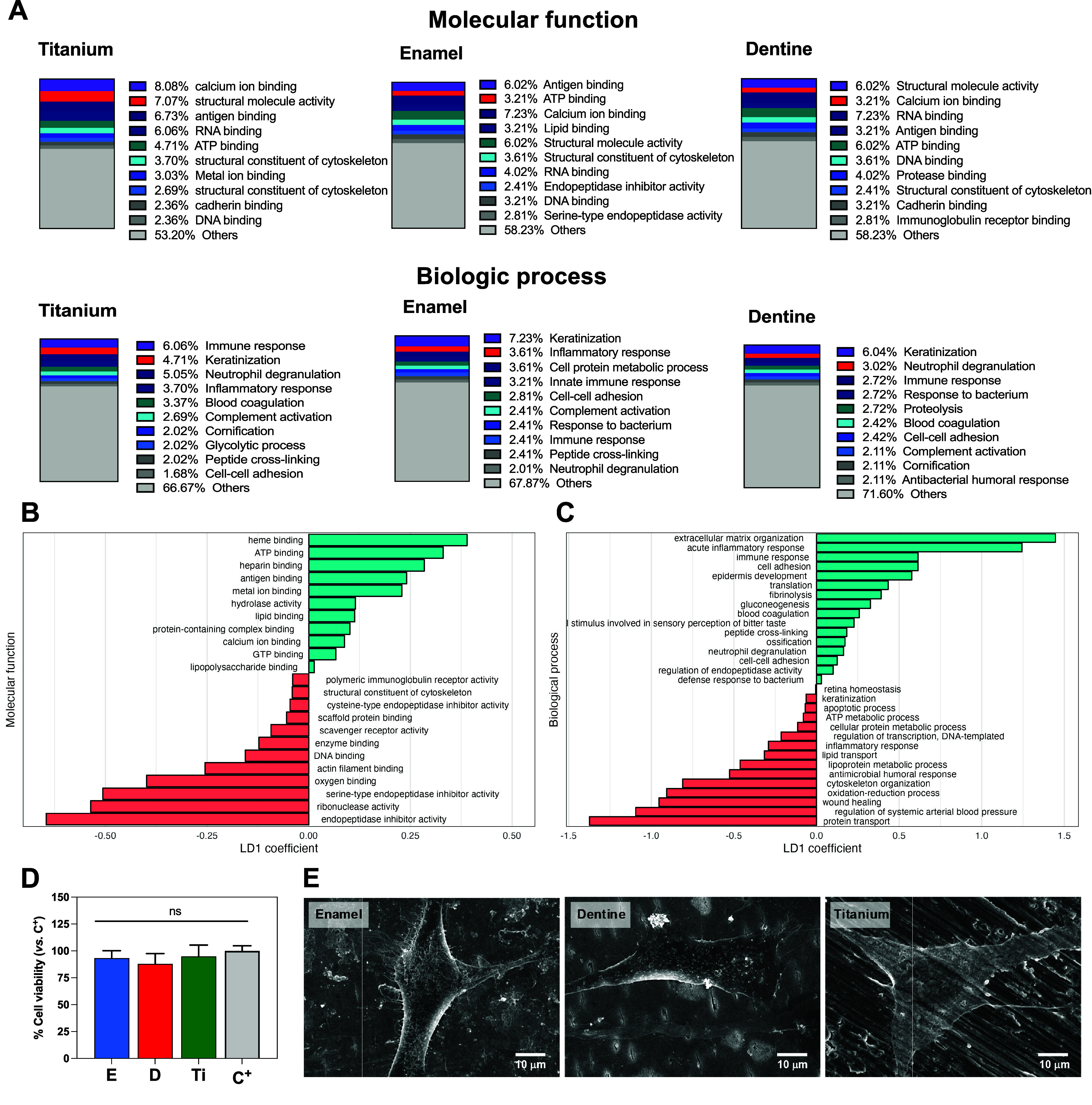
Molecular functions and biological processes mediated by the proteins identified in each substrate. (A) Top 10 molecular functions and biological processes with the highest number of proteins adsorbed onto each substrate. (B) Linear discriminant analysis (LDA) of the molecular functions that discriminate natural (dentine and enamel) and artificial (Ti) substrates. (C) LDA analysis of the biological processes discriminating natural (dentine and enamel) and artificial (Ti) substrates. (D) Viability (%) of MC3T3-E1 cells after 24-h incubation on different substrates (E = enamel, D = dentine, and Ti = titanium), expressed relative to the positive control (C+=standard culture plate surface). (E) SEM micrographs showing cell adhesion and morphology on the respective surfaces. Scale bar = 10 μm.

Considering the important biological events following salivary pellicle adsorption, we also evaluated cell adhesion and proliferation on salivary pellicle-coated substrates using MC3T3-E1 cells over 24 h. Cell viability (%) showed no significant differences among the substrates for this key biological parameter, with all salivary-coated surfaces reaching nearly 100% viability (Figure 4D). Scanning Electron Microscopy (SEM) revealed substrate-dependent variations in cell morphology (Figure 4E). On enamel surfaces, cells exhibited an elongated shape with prominent pseudopodia, suggesting active interaction with the surface. Cells also appeared elongated but more stretched on dentin, indicating favorable adaptation to the substrate. In contrast, cells on Ti showed a flatter and more spread morphology, typical of osteoblastic cells on Ti, which reflects strong adhesion and extensive surface contact.

### Substrate Properties Alter Bacterial Attachment and Biofilm Accumulation

Building upon the distinctions observed in salivary pellicle proteomes and their inferred molecular functions and biological processes mediated by proteins adsorbed from saliva arising from different surface properties, we evaluated whether these changes also modulated bacterial attachment and biofilm formation. To mimic an *in vivo* biofilm development, salivary pellicle was adsorbed for 2 h (time of *in vivo* salivary pellicle maturation) before testing microbial accumulation. To prevent bacterial attachment from initiating simultaneously with the protein adsorption phase, filtered human saliva was used. It is well-known that saliva processing methods do not significantly change saliva proteomic and microbial adhesion following pellicle formation.^33^ After this, the stimulated human saliva of healthy volunteers was used as a polymicrobial inoculum to initiate the oral microbiome binding to the substrates. To explore the patterns of similarity and dissimilarity among the samples for 2 h of microbial adherence, principal coordinate analysis (PCoA) was used. This revealed that Ti samples harbored a microbiome community that differed from that of enamel and dentine, as these latter two samples showed closer proximity to each other when compared with Ti (Figure 5A). However, certain enamel samples exhibited some proximity to the Ti samples, contrasting with the dentine samples, which displayed a more dispersed distribution. In terms of richness, alpha diversity by Shannon Index analysis showed no difference among the substrates (Figure 5B), suggesting that the number of species found in each group was similar. A total of 336 amplicon sequence variants (ASVs) were assigned a taxonomy at the bacterial species level for all substrates. Regarding the top-ranked nine most abundant species, most samples showed *Serratia marcescens* and *Rothia mucilaginosa* as the species with a higher proportion (Figure 5C). However, we also observed *Gemella sanguinis, Granulicatella adiacens*, *Haemophilus parainfluenzae, Porphyromonas pasteri, Pseudomonas fluorescens, Streptococcus parasanguinis,* and *Streptococcus salivarius* in all samples in smaller proportions. Surprisingly, only dentine had higher numbers of *P. fluorescens*. The Kruskal–Wallis test detected substrate-discriminating microbes at 2 h with standardized discriminant coefficients (Figure 5D). These findings underscored a closer similarity between enamel and dentine, with both substrates differentiating from Ti as far as microbiome composition on LDA was concerned. The abundance values of main bacterial species differentiating the substrates, species arrows closer to substrate centroid, were *Streptococcus parasanguinis* (HMT-411) for dentine; *Fusobacterium nucleatum* (HMT-202), *Prevotella oris* (HMT-311), and *Serratia marcescens* (HMT-115) for enamel; *Schaalia odontolytica* (HMT-701) and *Gemella sanguinis* (HMT-757) for the Ti surface (Figure 5D). A total of 30 bacterial species showed significant differences (*p* < 0.01) for abundance level values among the substrates (Figure 5E). The following species showed higher abundance for the Ti surface, compared with dental surfaces: *Cloacibacterium normanense* (HMT- 422), *Gemella sanguinis* (HMT-757), *Haemophilus parainfluenzae* (HMT-718), *Neisseria perflava* (HMT-101), *Neisseria subflava* (HMT-476), *Rothia mucilaginosa* (HMT-681), *Streptococcus australis* (HMT-073), *Streptococcus cristatus* (HMT-578), *Serratia marcescens* (HMT-115), *Schaalia odontolytica* (HMT-701), *Streptococcus oralis* (HMT-398), *Streptococcus parasanguinis* (HMT-411), and *Streptococcus sp*. (HMT-074) (Figure S3 - Microbes at 2 h that differ by substrate using Kruskal–Wallis test). As seen in PCoA analysis, two enamel samples showed some hierarchical proximity to the Ti samples. The differential tree showed that Ti and dental surfaces (enamel and dentine) were differentiated in terms of ASV counts in pair comparisons, irrespective of the statistical differences (Figure 5F). Hence, there are significant differences between dental and Ti surfaces in terms of microbiome profile during initial bacteria attachment (2 h) when exposed to human saliva. Thus, surface properties modulated the first biological response in the human body, namely protein adsorption, and a subsequent biological process, microbial adhesion. Given the ongoing concern regarding antimicrobial-resistant pathogens and their role in biofilm-induced diseases, we also tested whether salivary pellicle and substrate type modulate the adhesion of Methicillin-resistant *Staphylococcus aureus* (MRSA) after 2 h of incubation. All substrates showed increased MRSA adhesion following salivary pellicle formation (Figure S4 - Methicillin-resistant *Staphylococcus aureus* adhesion), which aligns with expectations, as protein adsorption is known to promote microbial attachment in the oral environment. Moreover, surface characteristics influenced the adhesion patterns. In the absence of a salivary pellicle, enamel exhibited the highest MRSA adhesion compared to dentine and Ti. However, following salivary pellicle formation, dentine showed the highest level of MRSA adhesion.

**Figure 5:**
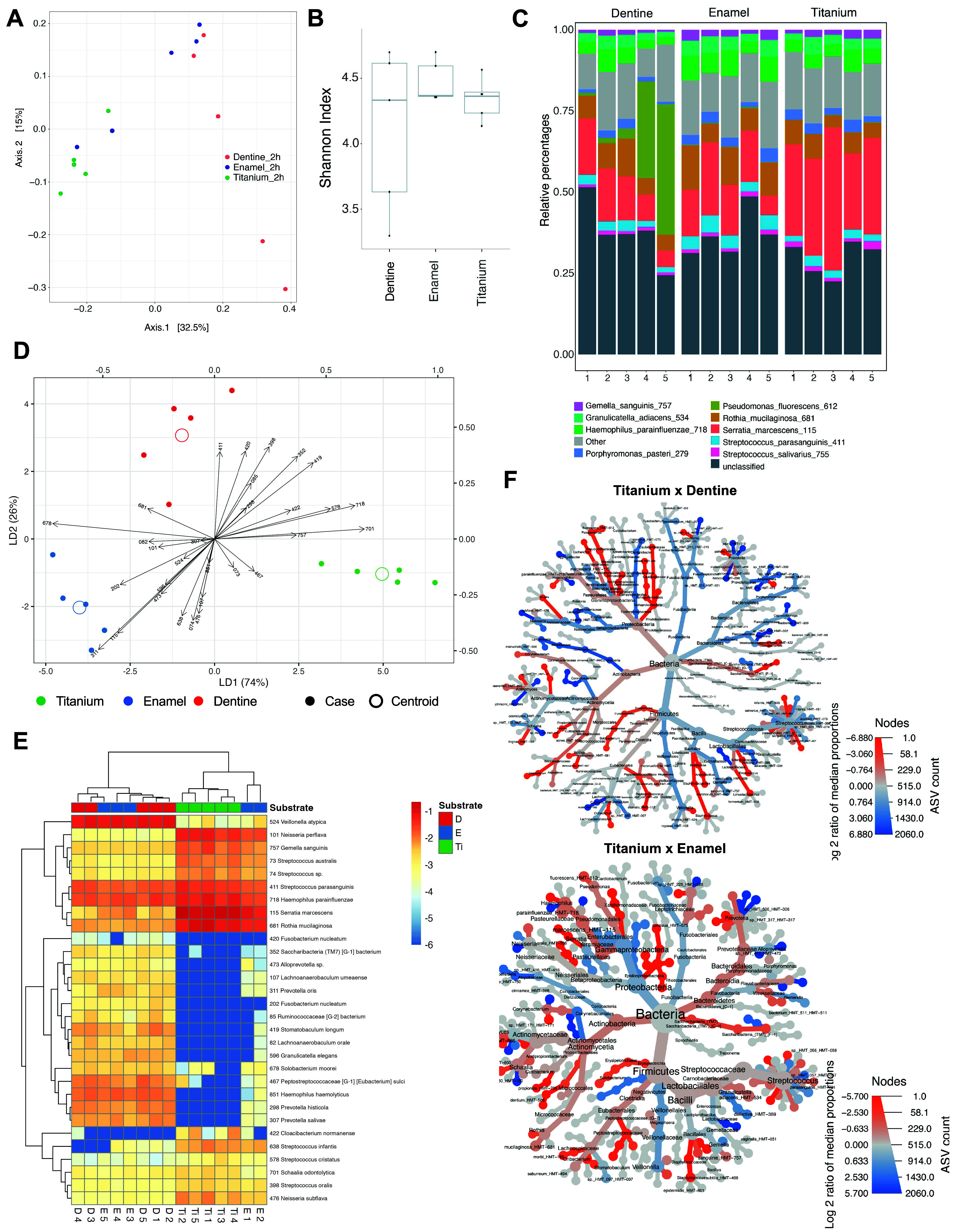
Microbiome analysis 2 h after microbial adhesion. (A) Principal coordinates analysis (PCoA) using the Bray–Curtis distance function and ASV abundance values. Microbial adhesion (2 h) was conducted using stimulated human saliva as microbial inoculum. The microbiome profile was evaluated by 16S RNA sequencing. (B) Alpha diversity analysis by the Shannon Index of sequenced samples from each substrate. (C) Stacking bar charts showing the relative abundance of species identified within sequenced samples. (D) Linear discriminant analysis (LDA) to quantify the discriminatory effect of each microbial species among the substrates. Kruskal–Wallis test detected substrate-discriminating microbes at 2 h with standardized discriminant coefficients. (E) Clustered heatmap of microbial species with a statistical difference (Kruskal–Wallis test - p-values below 0.01) in terms of ASV abundance among the substrates. (F) Differential heat trees comparing ASV abundance values between Ti and dental surfaces (dentine or enamel). Color indicates the dominant species in each substrate, Ti (red) or dental surfaces (blue), on a log2 median proportion scale. Node size indicates normalized ASV counts. D - dentine; E – enamel; Ti – Titanium.

Microbiome changes were observed after 24 h of biofilm formation. PCoA demonstrated that Ti surfaces harbored distinct microbiome communities compared to enamel and dentine, although dental surfaces showed a more diverse display (Figure 6A). There was no discernible difference across the substrates in terms of alpha diversity, with a slightly increased richness observed for the Ti surface (Figure 6B). Species such as *Streptococcus salivarius, Veillonella atypica,* and *Fusobacterium periodonticum* emerged as the main dominant species across all surfaces (Figure 6C), and a few new species showed increased abundance that was not there before binding (Figure 6C). The Kruskal–Wallis test detected substrate-discriminating microbes at 24 h, with standardized discriminant coefficients, revealed distinct microbiome profiles for all three substrates, with Ti exhibiting greater dissimilarity compared with both natural surfaces (Figure 6D). *Oribacterium parvum* (HMT-934) was the bacterial species closest to the dentine centroid, differentiating this substrate from the other two, in terms of species abundance (Figure 6D). Discriminant analysis indicated that *Prevotella nanceiensis* (HMT-299) was closer to the enamel centroid, and *Streptococcus intermedius* (HMT-644) and *Leptotrichia hongkongensis* (HMT-213) were closer to the Ti centroid (Figure 6D). A total of 23 bacterial species showed significant differences (p-values below 0.01) in terms of abundance level values among the substrates (Figure 6E). Importantly, remarkable changes were observed for the biofilm microbiome between 2 and 24 h (Figure 6F). When we explored the top 10 (highest changes) bacterial species in terms of increased or reduced abundance level over 24 h, we observed important fluctuations compared with the time interval of 2 h, indicating a potential microbiome shift after the binding phase. Notably, each substrate exhibited a different microbiome profile for the most increased and reduced bacterial species. In fact, PCoA analysis and alpha diversity showed important differences between the time intervals of 2 and 24 h for all substrates (Figure S5 - Microbiome analysis of 2 and 24 h after microbial adhesion and initial biofilm formation). This underscores the pivotal influence of substrate properties and salivary pellicle proteomics in shaping initial microbial accumulation.

**Figure 6:**
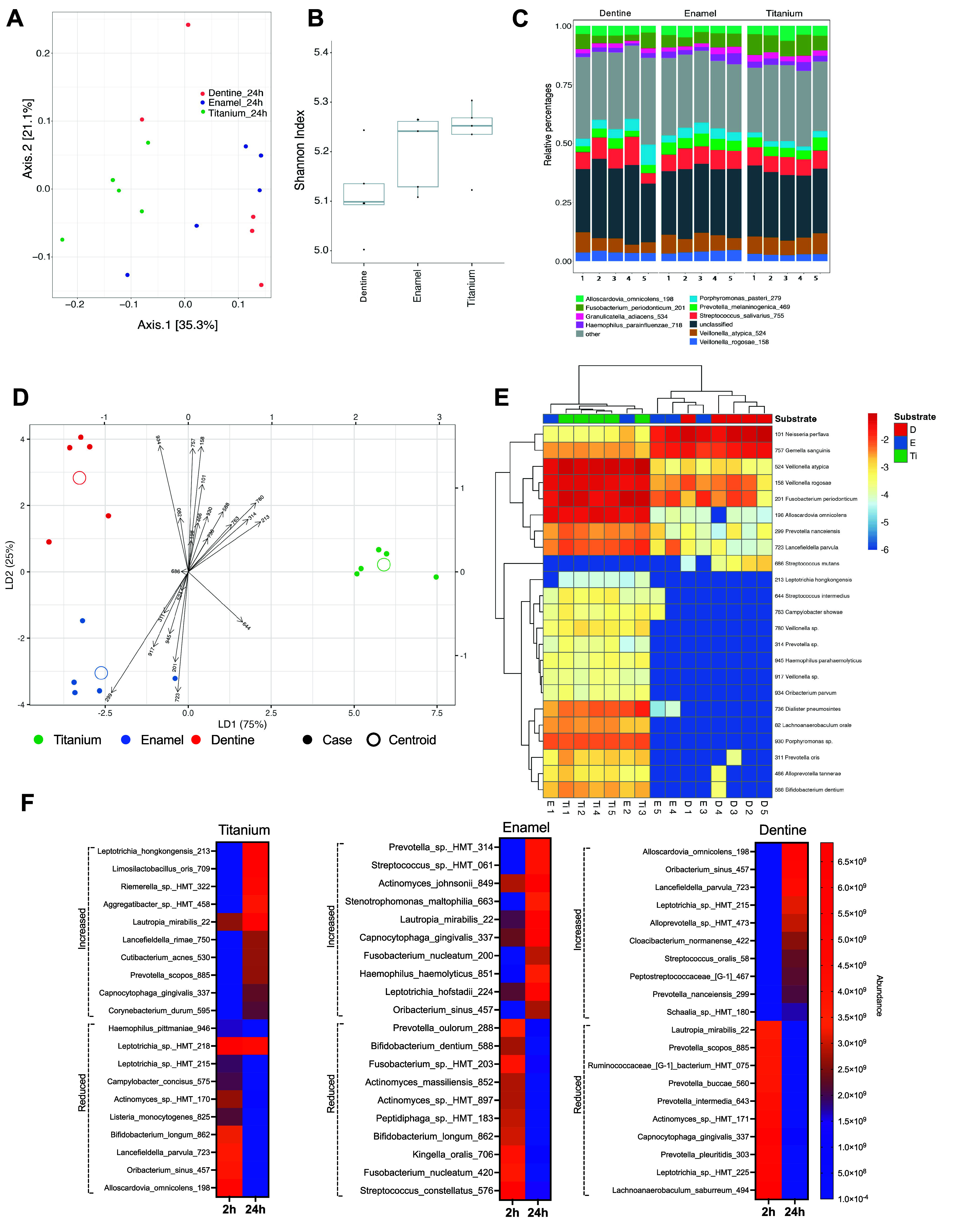
Microbiome analysis of initial biofilm formation (24 h). (A) Principal coordinate analyses (PCoA) using the Bray–Curtis distance function and ASV abundance values. Initial biofilm formation (24 h) was conducted using stimulated human saliva as microbial inoculum. The microbiome profile was evaluated by 16S RNA sequencing. (B) Alpha diversity analysis by the Shannon Index of sequenced samples from each substrate. (C) Stacking bar charts showing the relative abundance of species identified within sequenced samples. (D) Linear discriminant analysis (LDA) to quantify the discriminatory effect of each microbial species among the substrates. Kruskal–Wallis test detected substrate-discriminating microbes at 24 h with standardized discriminant coefficients. (E) Clustered heatmap of microbial species with a statistical difference (Kruskal–Wallis test - p-values below 0.01) in terms of ASV abundance among the substrates. (F) Heatmap showing the top ten bacterial species with higher increased or reduced abundance between 2 and 24 h of biofilm formation by substrate. D - dentine; E – enamel; Ti – Titanium.

### Proteome Profiles Were Correlated to the Attachment and Accumulation of Specific Bacterial Species

Given the high variation in the composition and level of proteomic and microbiome data, along with identifying proteins and bacterial species that differentiate the substrates, we used canonical correlation analysis (CCA) to identify highly associated levels of protein and microbial species. CCA obtains weighted sums of two sets of variables that are maximally correlated, from which we inferred the proteins that promoted or inhibited the adherence of which microbes. Additionally, our aim was to underline how microbe abundances during the initial adhesion phase (2 h) influenced their abundance in the subsequent biofilm formation (24 h).

Because the protein intensities and the microbial abundance values were measured from replicates from different samples, they were not paired in a manner in which each replicate served as a unit of observation. To obtain the most accurate estimate for each protein intensity or microbial abundance, we calculated the average across the replicates. First, we correlated protein enrichment and bacterial adhesion (2 h) data. Rather than mean values, we used the bootstrapped pairs data to calculate and visualize Pearson correlation coefficients. The classical Pearson correlation coefficient measures the linear relationship between the two variables and, therefore, distinguishes between positive and negative relationships. Figure S6 (Canonical correlation analysis to correlate protein and microbial species) shows the correlations between matched-substrate bootstrapped samples of protein levels and microbe abundances at 2 h.

Then we used our CCA models to conduct our tests. By distributing the variance (squared canonical correlation) in each canonical dimension onto the canonical coordinates of the protein levels, we obtained the interset correlations of the proteins and the intraset correlations of the microbes. Qualitatively, the vectors are closely aligned (or misaligned) when the correlation is strong and positive (or negative), and the vectors are roughly perpendicular when the correlation is weak. Longer vectors indicate species whose variation in the data is faithfully represented in the plot, and their correlation estimates are potentially larger as a result. Figure 7A shows the CCA results correlating protein and microbe data at 2 h. For example, the level of Annexin A2 (P07355–2) protein is closely related to the level of *Fusobacterium nucleatum* on the dentine substrate. Dermcidin protein (P81605–2) level is more closely associated with the level of *Haemophilus parainfluenzae* on enamel. Anexin A5 protein (P08758) level is more closely related to the level of *Streptococcus cristatus* on Ti. We used the same approach to correlating proteomic data with microbial data at 24 h to infer promoting and inhibitory relations between proteins and initial biofilm formation (Figure 7B) and microbial data at both time points (2 and 24 h), to infer how biofilm formation was affected by initial bacterial attachment (Figure 7C). The closest correlation (>0.7) between protein and microbial data, irrespective of the substrate, was described in the supplemental results (Tables S2 and S3 - Canonical correlation analysis to correlate protein and microbial species at 2 and 24 h) for all time points. The chord diagram displayed the inter-relationship between microbial species at 2 h and proteins with a high level of correlation (>0.7), which showed that the majority of bacterial species were correlated to more than the presence/intensity of one protein (Figure 7D). When we explored these correlations exceeding 0.7, we were able to visualize distinct relationships between certain microbial species and proteins, showing positive or negative correlations (Figure 7E). Evaluation of the top 3 instances of stronger positive and negative correlations between proteomes and microbiomes at the time interval of 2 h revealed that the presence or intensity of a specific protein promoted or impaired microbial adhesion (Figure 7F). For the top 3 stronger negative correlations (red background graphs), for example, the presence and higher intensity of Apolipoprotein E (P02649) protein reduced the adhesion of *Streptococcus parasanguinis* for dentine and enamel surfaces; but the absence of this protein on Ti substrate, allowed the higher adhesion of *S. parasanguinis* (Figure 7F). Among the top 3 stronger positive correlations (white background graphs), higher intensity of pancreatic adenocarcinoma up-regulated factor protein (Q96DA0) on Ti appeared to promote higher *Schaalia odontolytica* adhesion on this substrate (Figure 7F).

**Figure 7:**
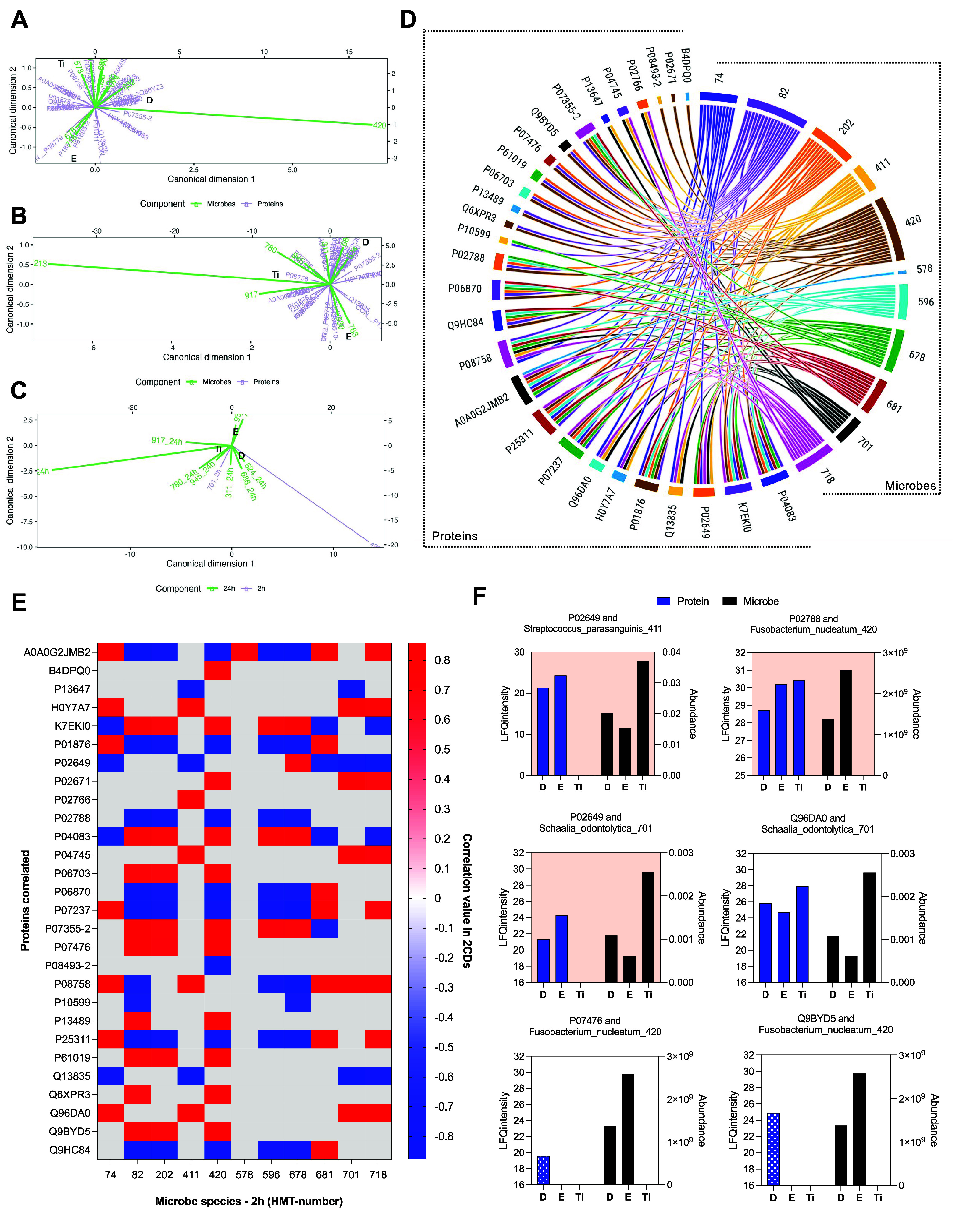
Canonical correlation analysis (CCA) to correlate protein and microbial species. (A) CCA analysis of proteomic data and microbial adhesion (2 h). Vectors are closely aligned (or misaligned) when the correlation is strong and positive (or negative), and the vectors are roughly perpendicular when the correlation is weak. Longer vectors indicate species whose variation in the data is faithfully represented in the plot, and their correlation estimates are potentially larger as a result. (B) CCA analysis of proteomic data and initial biofilm formation (24 h). (C) CCA analysis of bacteria adhesion (2 h) and initial biofilm formation (24 h). (D) Chord diagram displaying the inter-relationship between bacterial species (2 h) and proteins highly correlated (>0.7) by CCA analysis. (E) Heatmap showing the bacterial species identified at 2 h and highly correlated (>0.7) to salivary pellicle proteomic by correlation values distribution. (F) Top 3 strongest negative (red background) and positive (white background) correlations between proteomic and microbiome at 2 h.

Moreover, the presence and intensities of some proteins identified on the salivary pellicle were also highly correlated (>0.7) to microbial abundance values at 24 h (Table S3 - Canonical correlation analysis to correlate protein and microbial species at 24 h). Interestingly, the level of some bacterial species at 2 h was closely correlated (>0.5) to the level at 24 h, such as the presence of *Schaalia odontolytica (*HMT-701) at 2 h and *Haemophilus parahemolyticus* (HMT-945) and *Prevotella oris* (HMT-311) at 24h (Table S4 - Canonical correlation analysis to correlate microbial species at 2 and 24 h). Building on the proteomic data identified in terms of presence and intensity across all substrates and CCA analysis, we could now present a geometric prediction. Specifically, certain proteome profiles from salivary pellicles may serve as predictors for the higher or lower attachment of specific bacterial species. Moreover, this attachment can potentially influence the accumulation of particular species after biofilm formation for 24 h.

## Discussion

Previous studies have delved into the proteomic profile of salivary pellicles adsorbed onto implant devices, focusing on how this profile is modulated by changes in surface topography, particularly on rough materials.^4,13,14,34^ Hence, it would be reasonable to infer that variation in surface properties impacts protein adsorption onto the substrates tested. In the case of initial salivary pellicle adsorption (3 min), similar patterns have been observed for enamel and Ti (99.3% Ti, 0.3% iron, 0.25% oxygen) according to proteomic analysis.^35^ Our study demonstrated that Ti salivary pellicle proteomes differ from those of enamel and dentine in terms of composition and intensities when exposed to whole simulated human saliva and after pellicle maturation over 2 h. Although the substrates showed 67% of shared proteins, surface properties led to the adsorption of certain specific proteins onto each substrate, mainly for dentine, which affected the main biological process mediated by the proteins adsorbed. These differences may be attributed to marked variations in surface properties, mainly in terms of chemical composition and physical properties. Moreover, our geometric statistical analysis identified specific proteins whose adsorption patterns effectively discriminate between substrates. We also compared Ti biomaterial and dental surfaces, focusing on polymicrobial attachment and initial biofilm formation. Although the substrates displayed some similarities in microbial communities, over 20 bacterial species showed significant differences among the substrates in abundance, and remarkable changes were found over 24 h. Importantly, we provided experimental and statistical modeling evidence, demonstrating a correlation between the presence and intensity of specific proteins and the adhesion of specific bacterial species. These findings offer essential insights into understanding the first biological responses after inserting implanted devices in the oral cavity. Additionally, they underscore differences between dental tissues and biomaterials during the initial steps of microbial colonization and the pivotal role of certain proteins in promoting or reducing the adhesion of specific bacterial species.

Despite differences in the chemical and physical properties of the tested substrates (Ti x dental surfaces), there was no significant impact on the overall number of proteins adsorbed onto the surfaces. However, there were some differences in the profile and intensity of these proteins. Identifying exclusive proteins for each substrate can enhance our understanding of biological processes specific to these substrates. For example, two Annexin (A4 and A5) proteins were exclusively identified on Ti, which is involved in cell migration and enhanced immunogenicity processes,^36^ important steps during the implant healing process. Importantly, discriminant proteins indicated which proteins differentiated each substrate and could be explored by biomedical engineering when developing novel surfaces or coatings for existing biomedical materials. An illustrative example is lactotransferrin, which serves as a discriminant protein distinguishing Ti from dental surfaces, according to our LDA. This protein, recognized by its antimicrobial effect, has been used as a coating for implant devices.^37^ Interestingly, lactotransferrin (P02788) was negatively correlated (>0.7) with five bacterial species at 2 h of adhesion (*Lachnoanaerobaculum orale, Fusobacterium nucleatum subsp. polymorphum, Fusobacterium nucleatum subsp. animalis, Granulicatella elegans*, and *Solobacterium moorei*). Therefore, this information holds useful promise for developing smart biomaterials to prevent, control, and treat biofilm-related infections, using these specific proteins as target agents to control microbial attachment. Importantly, in this study, we reproduced a biological state representative of healthy individuals, minimizing the influence of disease and physiological variability during saliva collection and in its composition. Therefore, the effects of various physiological factors (e.g., pH, age, and diet) and, in particular, pathological conditions (e.g., implant-related infections), both systemic and oral, should be further explored in future studies. This will help to understand how these variables impact the formation and composition of the salivary pellicle on biotic and abiotic surfaces, as well as their influence on microbiome signatures—contributing to a broader understanding of these processes within a heterogeneous patient population.

The immune response was outstanding as a pivotal step for the long-term survival and healing of tissues surrounding implant devices and emerged as the primary biological process identified for proteins adsorbed onto Ti.^38^ Moreover, the inflammatory process was a biological process that differentiated Ti from dental surfaces by LDA. Previous evidence has compared immune response related to peri-implantitis (implant surface) and periodontitis (dental surface) diseases, showing increased immune cell densities for implant devices,^39^ elevated proportions and activation of macrophages,^40^ and up-regulated cyclooxygenase-2 pathway.^41^ Our results may suggest a significant mechanism that explains the heightened immune response observed for implant devices. Identifying the immune response as the predominant biological process for proteins adsorbed onto Ti suggested that this substrate had a high affinity for proteins acting in immune response processes. On the Ti surface, 34 proteins were identified to play a role in immune and inflammatory responses, compared with 21 for dentine and 23 for enamel. Notably, among the exclusive proteins detected, only the Ti substrate exhibited an immunoglobulin (specifically, immunoglobulin kappa variable 2–30), distinguishing it from the exclusive proteins found on dental surfaces. Another example only found on Ti was coronin 1A, associated with immune responses, and previously identified on rough Ti surfaces.^42,43^ Interestingly, the proteome of peri-implant crevicular fluid has shown a high number of proteins related to humoral immune response, response to bacteria, and response to external stimuli.^44^ Some of these proteins previously found on peri-implant crevicular fluid and related to immune response were also found on Ti in our study (i.e., P17213, A0A0A0MTS2). Further studies targeting improved implant surfaces should delve deeper into this finding to enhance biological responses and mitigate inflammatory responses, even without microbial communities. Moreover, immune-related proteins may be useful in discussing the role of host immune response in implant healing and osseointegration processes, considering that aseptic inflammatory processes have been reported for orthopedic implants.^45^

Biofilm accumulation in the oral environment has been considered the main villain in inducing prevalent diseases in the oral cavity, including periodontitis on dental surfaces and peri-implantitis on dental implants.^46^ Any surface exposed to the oral environment is immediately coated by proteins, subsequently facilitating bacterial attachment. As anticipated, surface properties affected the salivary pellicle proteomic profile, thereby impacting the initial bacterial attachment at 2 h. Although the initial shapes of the adhesion profile of biofilms developed after 24 h, bacterial growth was also affected by specific environmental conditions, such as medium, oxygen level, and carbohydrate sources, of which tests were conducted under specific and static conditions in our study. Although periodontal disease and dental implant-related infections exhibited similar microbial compositions,^47^ differences have been observed in the coaggregation process, microbial proportion, and succession. *In vivo*, evidence has also shown similar patterns and levels for initial microbial adhesion (30 and 120 min) for dental and Ti surfaces, mainly in terms of the attachment of *Streptococcus spp*. and *Actinomyces* species.^48^ Clinical-induced gingivitis and peri-implant mucositis have shown similar abundances and community structure for both substrates at baseline; however, after oral hygiene abstention, a consistent shift in global microbiome communities was observed for tooth surfaces but not for implants.^49^ In fact, our results showed differences among the substrates for bacterial species that increased and reduced during biofilm growth (after 24 h). Moreover, recent insights into dental implant-related infections by means of strain-resolution metagenomics have suggested the involvement of new pathogens, including *Prevotella intermedia*, *Fretibacterium fastidiosum,* and *Fusobacterium nucleatum,* expanding the spectrum beyond the well-known red microbial complex associated with tooth surfaces.^50^ Here, we identified some differences and discriminant bacterial species between Ti and dental surfaces at 2 and 24 h. Understanding the initial steps of bacterial attachment and microbial accumulation highlights the differences between biotic and abiotic surfaces, even when exposed to the same environment. Commonly, knowledge gained from dental surfaces in terms of microbiological findings has been extrapolated to implant devices without experimental validation. However, the discriminant proteins and microbial species identified in our study emphasize that these substrates behave differently in the oral environment, mainly in terms of salivary pellicle formation and bacterial adhesion profile.

Our geometric approach identified proteins closely correlated to specific bacterial species, irrespective of the substrate. However, leveraging our proteomic data enabled us to discern whether these proteins exhibit high adsorption on the substrates tested. Eleven (11) bacterial species were highly (>0.7) correlated to several proteins. The same bacterial species was correlated to one or more proteins. Importantly, certain correlation pairs (microbe and protein) exhibited positive or negative correlations, indicating that the presence and/or intensity of certain proteins promoted or reduced microbial adhesion (2 h). These correlations matched the proteomic and microbiome data found. Negative correlations suggested that the absence of certain proteins resulted in higher specific microbial adhesion or increased intensity of specific proteins, which led to reduced microbial adhesion. Conversely, positive correlations indicated that the absence of certain proteins also led to the absence of certain bacterial species, or increased intensities of specific proteins resulted in increased attachment of certain bacterial species. Apolipoprotein E protein was negatively correlated to *Streptococcus parasanguinis,* being the strongest correlation (>0.8) found. Based on our data, a higher intensity of Apolipoprotein E protein reduced the adhesion of *Streptococcus parasanguinis* on dentine and enamel surfaces. Moreover, the absence of this protein on Ti allowed for higher adhesion of the same bacterial species. Interestingly, this protein and its derived peptides have been documented relative to exhibit significant antibacterial activity, particularly against pathogens such as *Pseudomonas aeruginosa* and *Escherichia coli*.^51,52^ Furthermore, the Apolipoprotein E protein is known to bind to bacterial lipopolysaccharide (LPS), thereby regulating the inflammatory process and modulating levels of proinflammatory cytokines.^51^ These findings provided significant biological plausibility for correlations found by our geometric analysis. The correlations identified hold significant potential for biomedical engineering applications, allowing for the development or enhancement of biomedical devices with targeted biological responses. For instance, promoting specific protein binding can increase the presence of health-associated bacterial species or suppress disease-associated types.^53,54^ The oral environment is densely colonized by microbial species, and preventing microbial adhesion poses clinical challenges. However, this knowledge can be harnessed to modulate microbial accumulation, control microbial shift, and foster resilience in microbial communities within the oral cavity. Although our correlation models were not linear, and different environmental and molecular factors can directly affect protein adsorption and bacterial attachment in the oral cavity, these findings provided useful information for future hypothesis-driven research. Moreover, the identified protein biomarkers can likely be used by biomedical research to predict further adhesion and accumulation of pathogenic species involved in implant-related infections and to develop novel biomaterial devices with enhanced biological responses.

Protein adsorption onto solid surfaces, such as Ti and dental surfaces, is governed by external factors, substrate properties, and intrinsic protein characteristics. Experimental and environmental conditions, such as temperature, pH, and the composition of the surrounding medium or buffer, can directly influence the adsorption process. In our study, these conditions were carefully controlled to mimic the oral environment. In complex biological fluids like saliva, protein adsorption involves a dynamic interplay of transport, adhesion, and repulsion mechanisms. As such, both the physicochemical properties of the substrate and the nature of the proteins influence adsorption outcomes. Notably, proteins tend to adsorb more readily onto nonpolar surfaces with high surface tension, as well as onto charged surfaces.^3^ The secondary and tertiary structures of proteins include side chains with diverse properties, including both positive and negative charges.^3^ Due to this structural complexity, a single protein may exhibit different affinities for different substrates. Moreover, external parameters can alter protein surface properties; for instance, at low pH, proteins tend to acquire a positive charge, while at high pH, they may become negatively charged.^55^ Beyond protein structure, substrate characteristics are key factors influencing the amount and composition of adsorbed proteins. Previous studies have shown that surfaces with greater irregularity, protrusions, hydrophobicity, and roughness typically promote increased protein adsorption^56^—a pattern also observed in our study for Ti and dentine, which exhibited a higher number of adsorbed proteins. This phenomenon is attributed to the increased surface area and availability of binding sites,^57^ as well as a higher affinity for proteins with specific conformations.^58^

Surface charge is another surface property that mediates protein adsorption, as electrostatic interactions play a key role in the adsorption process. Ti substrates are typically negatively charged, as demonstrated by ζ-potential measurements,^59^ which may result in the repulsion of negatively charged proteins. Although most salivary proteins carry a net negative charge, protein structures are highly heterogeneous, particularly in charge distribution.^60^ As a result, even negatively charged proteins can adsorb onto negatively charged surfaces due to localized surface and protein charge variations. Importantly, previous evidence has shown that modifying the surface charge of Ti to make it more positive can enhance protein adsorption and promote subsequent human cell adhesion.^61^ This strategy has been widely employed in biomedical engineering to promote favorable biological responses. However, most studies have focused on individual proteins’ adsorption behavior. In contrast, our findings demonstrate that, within a complex protein mixture such as saliva, there is no significant difference in the overall amount or composition of adsorbed proteins when comparing Ti to tooth surfaces. Notably, both enamel and dentine also exhibit a negative surface charge, even after salivary coating,^62^ which may explain the similar protein adsorption patterns observed across these substrates.

Although surface charge and topography are important mediators of protein adsorption, the chemical composition of substrates plays a crucial role in determining both the amount and conformation of adsorbed proteins. The presence of specific functional groups on the substrate surface, such as – OH or – CH_3_, can influence the nature and strength of protein–surface interactions, as well as the resulting protein conformational changes.^63^ Elements intrinsic to the surface composition can further modulate the selectivity and structural conformation of absorbed protein—for instance, the calcium and phosphate present in enamel and dentine due to their hydroxyapatite content.^64^ Calcium, in particular, has a strong affinity for protein binding and is highly abundant on tooth surfaces.^65^ While direct binding between calcium ions and Apolipoprotein E has not been demonstrated, this protein has been shown to induce the release of intracellular free Ca^2+^,^66^ suggesting potential interactions that warrant further investigation through binding assays. Moreover, since Apolipoprotein E is negatively charged,^67^ adsorption onto tooth surfaces may be facilitated by the presence of positively charged calcium ions—an interaction likely absents on Ti, which may explain its reduced affinity for this protein.

Importantly, although we did not evaluate the effect of surface properties and proteomic signatures on the total amount of biofilm accumulation, this aspect has already been addressed by previous evidence. Both in vitro and in vivo evidence have highlighted greater biofilm accumulation on dental surfaces than on Ti.^16,26,48^ Therefore, the focus of our study was to shed light on how surface properties and proteomic signatures shape microbial composition during early biofilm adhesion, providing new insights into this key biological response. Since proteins adsorbed onto surfaces act as a bridge for microbial adherence, the protein profiles identified in this study, including the discriminant proteins among the substrates and their correlations, provide important insights into the relationship between proteins and bacteria on dental and implant surfaces. Based on our findings and other studies demonstrating that surface properties modulate microbial adherence and interactions, these outcomes cannot be simply transferred to other biomaterials. Changes in surface properties need to be evaluated in terms of molecular responses within biological systems, such as the human body. However, here we highlight the impact of surface properties on two important biological responses: protein adsorption and bacterial adhesion. Our results show that even when these processes occur in the same environment, they are modulated by the substrate.

## Conclusion

Thus, our findings showed that differences in surface properties drive distinct changes in the salivary proteomic profile, differentiating dental surfaces from Ti materials. These differences subsequently modulate polymicrobial attachment and accumulation. Moreover, we identified for the first time strong correlations between specific proteins and bacterial species according to the substrate. These insights can be further explored by biomedical engineering to develop new surfaces that promote favorable biological responses and prevent microbial attachment. Further studies are needed to investigate the impact of surface properties on different microbial kingdoms, such as fungi, changes in microbial pathways at the gene level, and biofilm structure, to uncover key properties that can be used to enhance current implant devices and improve clinical outcomes.

## Methods

### Ethical Aspects

This study was approved by the local Research and Ethics Committee (protocol 55366416.0.0000.5418). Stimulated human saliva was used for salivary pellicle formation and biofilm assay. Saliva was collected from 8 healthy volunteers according to previously described inclusion criteria: over 18 years old, good systemic and oral health, normal stimulated salivary flow rate (>0.7 mL/min), no recent antibiotic intake within the month prior to the study, being nonsmokers, and not using mouthwash.^68,69^

### Substrates

Commercially pure Ti (cpTi) discs grade II (o̷ = 8 mm × 2 mm) (Conexão Ltd., São Paulo, Brazil) were sequentially polished (roughness average, Ra 0.20 ± 0.06 μm) and used as a substratum for proteomic and microbial assays. Bovine teeth, which have been widely used as a substrate to evaluate oral diseases due to their similarity to human teeth composition,^68^ were used to prepare 4 × 4 × 2 mm enamel slabs. Polished root dentine slabs (7 × 4 × 1 mm) were also obtained from bovine incisors.^70^ Substrates were ultrasonically cleaned with deionized water and 70% alcohol (10 min) and sterilized by ultraviolet light (20 min) prior to use.^71^ Substrates were characterized by a 3D laser scanning confocal microscope (LSCM) (VK-X200 series, Keyence, Osaka, Japan) to acquire three-dimensional images using lenses of 50× and 150× magnifications for surface topography observation.^72^ Images were processed using VK analyzer software (Keyence v3.3.0.0, Osaka, Japan). Atomic force microscopy (AFM) (Park NX10; Park System, USA) was employed to evaluate both 2D and 3D surface topography in tapping mode, using a silicon probe under controlled environmental conditions (25 °C ± 1 °C; 5% ± 1% humidity). Scans were performed over a 30 μm × 30 μm area, producing high-resolution images with 512 × 512 pixels for detailed surface analysis. The chemical composition of surfaces was assessed by X-ray photoelectron spectroscopy (XPS; Vacuum Scientific Workshop, VSW HA100, Manchester, U.K.) following the parameters previously described.^73^ Deionized water contact angle measurements were performed using the sessile drop method with an automated goniometer (Ramé-Hart 100–00; Ramé-Hart Instrument Co., Succasunna, USA) and analyzed with DROPimage software. Contact angles were calculated as the average measurements taken from both sides of 5-μL water droplets placed on the sample surfaces. Physicochemical and topography analyses were used to explore the differences between the substrates.

### Saliva Collection

A pool of fresh stimulated human saliva from eight volunteers with good systemic (no self-reported systemic condition) and oral health (absence of dental caries, periodontal and peri-implant diseases after clinical examination) and a normal stimulated salivary flow rate (>0.7 mL/min) was used.^72,74^ The saliva was donated at least 2 h after the volunteers had eaten and brushed their teeth, and saliva flow was stimulated by chewing a piece of flexible film (Parafilm M; American Can Co., Neenah, WI, USA). A pool of saliva was used fresh immediately after collection and kept on ice during assays.

### Salivary Pellicle

To mimic the *in vivo* dynamic of protein adsorption and to avoid high-molecular-weight protein loss,^33^ the pool of saliva was not filtered but centrifuged (10 min, 3,800 g) to remove cell debris or other contaminants. Then, substrates were immersed in 1 mL of saliva in a 24-well plate for 2 h (maximum maturation of in vivo salivary pellicle) at 35 °C in an orbital shaker (70 rpm).^75^ After protein adsorption, samples were washed (3×) with saline solution (0.9% NaCl) to keep only proteins adsorbed onto the surfaces, and 10 samples of each group were pooled in a polypropylene tube containing 10 mL of purified water to be used later for protein elution.^12^

### Liquid Chromatography Coupled with Tandem Mass Spectrometry (LC–MS/MS)

Protein elution was managed according to a previous protocol.^76^ Samples were vortexed for 1 min, sonicated at 7 W (4 °C) for 5 min (model S-150D; Branson Ultrasonics Corp., Danbury, CT, USA), vortexed for 1 min, and stored at −80 °C.[13] Then, samples were lyophilized and resuspended in 50 μL of urea (8 M). Total protein quantification was conducted using the Bradford method, with bovine serum albumin (BSA) as the standard.^77^ The proteins were reduced, alkylated, and digested by trypsin (1:50, w/w), and the proteins extracted were subjected to LC–MS/MS.^12,14^ Samples were dried in a vacuum concentrator and reconstituted in 22.5 μL of 0.1% formic acid. An aliquot of 4.2 μL (0.88 μg) of peptides was analyzed on an LTQ Orbitrap Velos mass spectrometer (Thermo Fisher Scientific, Waltham, MA, USA) connected to the EASY-nLC system (Proxeon Biosystem, West Palm Beach, FL, USA) by means of a Proxeon nanoelectrospray ion source. Peptides were separated by a 2–90% acetonitrile gradient in 0.1% formic acid in an analytical PicoFrit Column (20 cm × ID75 μm, 5 μm particle size) (New Objective Inc., Woburn, MA, USA) at a flow rate of 300 mL/min over 80 min.^33,78^ All instrument methods were set up in the data-dependent acquisition mode. The full-scan MS spectra (*m*/*z* 300–1,600) were acquired in the Orbitrap analyzer after accumulation to a target value of 1 × 10^6^. The resolution in the Orbitrap was set to r = 60,000, and the 20 most intense peptide ions with charge states ≥ 2 were sequentially isolated to a target value of 5,000 and fragmented in the linear ion trap by low-energy CID (normalized collision energy of 35%). The signal threshold for triggering an MS/MS event was set to 1,000 counts. Dynamic exclusion was enabled with an exclusion size list of 500, a duration of exclusion of 60 s, and a repeat count of 1. An activation q = 0.25 and an activation time of 10 ms were used.^79^ Each sample was subjected to three readings. Peptide sequences acquired were identified using MaxQuant software (v.1.3.0.3), and MS/MS spectra were searched against the Human UniProt database (released in May 2017; 92,646 sequences; 36,874,315 residues). Perseus v.1.5 software using the LFQ algorithm was used for protein intensities/quantification, and the list of peptides identified was filtered by a minimum localization probability of 0.75, and reverse and contaminant entries were excluded from further analysis. Venn diagrams were constructed using a free web-based tool^80^ to compare the substrates; molecular function, biological process, and mass (Da) of proteins identified were checked and extracted manually from the UniProt database.

### Microbial Adhesion and Initial Biofilm Formation

Different experiments were conducted to evaluate the effect of substrates and salivary pellicle proteomes on bacterial adhesion (2 h) and initial biofilm formation (24 h). First, substrates were exposed to salivary pellicle formation using stimulated human saliva from the same 8 healthy volunteers mentioned above. However, for salivary pellicle, the pool of saliva was centrifuged (10 min, 3,800 *g*) and filtered (0.22 μm poly­(ether sulfone) membrane, JET Biofil syringe filter) to remove microbial cells. Our previous study showed that a 0.22 μm filter slightly changed saliva proteomes, and more than 75% of proteins identified in nonprocessed saliva were kept after the processing method (0.22 μm filter).^33^ Importantly, we have previously shown that different saliva processing methods did not affect some microbial species’ adhesion.^33^ After 2 h of salivary pellicle formation (35 °C, 70 rpm, 1 mL per well), samples were washed (3×) with 0.9% NaCl to remove detached proteins. Thus, protein-coated substrates were transferred to new wells containing Brain Heart Infusion (BHI) media and nonprocessed stimulated human saliva (1:10 v/v) as microbial inoculum and incubated for 2 (adhesion) or 24 (initial biofilm) hours, under static conditions at 37 °C in a 5% CO_2_ incubator.^74^ Biofilms were collected by sonication, centrifuged to pellet the biofilm for subsequent microbiome analysis.^74^ All experiments were conducted at least in duplicate.

### Genomic DNA Isolation and Illumina Sequencing

Genomic DNA was extracted from pellets using the ZymoBIOMICS-96 MagBead DNA Kit (Zymo Research, Irvine, CA) by an automated platform. DNA quantity and quality were evaluated by absorbance (A260/A280) values using NanoDropOne/One C (Thermo Fisher); quantification and amplification by RT-PCR OPUS (Bio-Rad); qualification of fragments during library preparation by 4200 TapeStation (Agilent); and pool quantification by Qubit 4 Fluorometer (Thermo Fisher). Bacterial 16S rRNA gene sequencing was performed using the Quick-16S NGS Library Prep Kit (Zymo Research, Irvine, CA). The V3–V4 region of the 16S rRNA gene was amplified. Final pooled libraries were cleaned with the Select-a-Size DNA Clean & Concentrator (Zymo Research, Irvine, CA) and then quantified with TapeStation (Agilent Technologies, Santa Clara, CA) and Qubit (Thermo Fisher Scientific, Waltham, WA). The final libraries were sequenced on Illumina MiSeq with a v3 reagent kit (600 cycles).

The 16S rRNA amplicon sequence variants (ASV) were inferred from raw reads using DADA2.^81^ ASV counts were normalized using Metacoder.^82^ ASV taxonomic assignment was performed using a previously described custom DECIPHER classifier^83^ generated from the 16S rRNA gene database of the Human Oral Microbiome Database (eHOMD).^22,84^ Alpha diversity metrics and beta diversity plots were generated using Metacoder. To compare differences between community members among samples, differential heat trees were generated using the Metacoder “heat_tree” function.

### Antimicrobial-Resistant Pathogen Adhesion

Given the growing concern over antimicrobial-resistant pathogens, a 2-h adhesion assay was performed to specifically assess the early colonization potential of a resistant bacterial strain on different substrates in the presence or absence of a salivary pellicle proteome. Methicillin-resistant *Staphylococcus aureus* (ATCC 33591) strain was cultured overnight and adjusted to 10^7^ cells/mL in BHI medium (OD = 0.1 at 550 nm). The substrates, either preconditioned with a salivary pellicle or left untreated, were incubated with the bacterial suspension (10:1 v/v in BHI) at 37 °C under 10% CO_2_ for 2 h to allow initial adhesion. Following incubation, samples were gently rinsed with 0.9% NaCl to remove nonadherent cells. Adherent bacteria were then recovered by sonication, serially diluted, and plated on BHI agar for colony-forming unit (CFU) quantification.^33^

### Cell Adhesion Effect

For cytocompatibility evaluation, murine preosteoblastic MC3T3-E1 cells were cultured on saliva-coated substrates to assess both cell viability and morphology. Cells were seeded at a density of 1 × 10^4^ cells per well in 48-well plates containing alpha-modified Eagle’s medium (Gibco, Thermo Fisher Scientific, Massachusetts, USA), supplemented with 10% fetal bovine serum and 100 U/mL penicillin-streptomycin. Cultures were maintained in a humidified incubator at 37 °C with 5% CO_2_. After 24 h of incubation, cell viability was quantified using the Alamar Blue assay (Thermo Fisher Scientific, Massachusetts, USA). For morphological analysis, samples were fixed with 2.5% glutaraldehyde, rinsed, and dehydrated through a graded ethanol series (35%, 50%, 70%, 90%, and 100%) at room temperature. The samples were then subjected to critical point drying, gold sputtering, and imaging using scanning electron microscopy (SEM) (JEOL JSM-6010LA; JEOL, Peabody, USA).

### Statistical Analysis

#### Roughness, Wettability, Cell Viability, and CFU Data

Statistical analyses were conducted using GraphPad Prism version 10.4.2 for macOS. One-way ANOVA was applied to evaluate differences in surface roughness, wettability, and cell viability across the substrates. For bacterial adhesion (CFU counts), a two-way ANOVA was performed considering substrate type and presence of salivary pellicle as independent factors. Tukey’s posthoc test was used for multiple comparisons. A significance level of α = 0.05 was adopted for all analyses. In the graphs, statistically significant differences are indicated by asterisks: *p < 0.05, **p < 0.01, ***p < 0.001.

#### Proteomic Data

To evaluate how protein levels varied across enamel, dentine, and Ti, we adapted a pipeline by Segata et al. (2011)^85^ to detect and characterize discriminants among these substrates. First, histograms of pooled amounts within each substrate were generated to assess their similarity and skewness. For all proteins not detected by LC–MS/MS in some substrate or specific reads, a constant intensity below the detection limit at each missing entry was imputed to include all proteins in the global analysis. For this, the imputed value was chosen to minimize the skewness of the total distribution, unbatched by substrate or repetition, as measured by Pearson’s second skewness coefficient (this value results in equality between the median and mean.) Then, a Kruskal–Wallis (KW) rank sum test was conducted for each protein across the three substrates. We discarded proteins for which the KW statistic yielded a p-value of 0.05 or higher. Then, the intensity data for these likely biomarkers was used to generate a clustered heatmap by substrate, where the row and column dendrograms encode complete-linkage hierarchical clusters based on Euclidean distances between measurements. The pipeline proceeded to use linear discriminant analysis (LDA) to quantify the ability of each protein to discriminate among the substrates. To measure variable effects, we reported standardized discriminant coefficients, which quantify the contribution of each variable to the discriminant scores after controlling for their variances. A two-dimensional biplot was generated to visualize the case scores together with within-substrate centroids, with the variable coefficients along the two discriminant axes. The greater the alignment between the position of a sample (or within-substrate centroid) and the direction of a protein vector, the greater the differential intensity of that protein in that sample (or substrate). In order to understand the differential protein signatures of the natural/biotic (dentine and enamel) and artificial/abiotic (Ti) substrates, we repeated the preceding analysis pipeline using this grouping of the samples.

A similar approach was used to identify differences in the molecular functions suggested by the levels of the proteins. Within each substrate (or group), a measure of the cumulative intensity of the proteins associated with each molecular function was computed, given by the sum of the intensities of its associated proteins. These measurements were then used in place of protein intensities to identify functions that discriminated among the substrates.

#### Microbial Data

We used the same analysis pipeline to analyze microbial abundance values as protein levels. However, microbial abundance values were extremely right-skewed and zero-inflated, so we used the offset logarithm x → log_10_(α + x) to rescale the data, with α obtained from the formula below, where [*q*] indexes the microbes and [*n*] indexes the cases:

$$\alpha =\exp_{10}\left(\left[\log_{10}\left(\min\left\{a_{ij}:i\in \left[n\right],j\in \left[q\right]\right\},\left\{0\right\}\right)\right]-1\right)$$

KW tests and LDA were performed in the same way as on the protein data, except that only microbes with *p* < 0.01 were included. An analysis of the biological processes implicated by the microbes observed was performed analogously to that of the molecular functions associated with the measured proteins.

#### Proteomic and Microbial Data – Correlation and Prediction Analysis

To analyze whether specific protein levels predicted microbe abundance values, we applied correlation analysis and canonical correlation analysis (CCA) to the protein and microbe data. Because the data were unpaired (collected from different experiments) and small (many more variables than cases), we facilitated these analyses by constructing a bootstrap sample: For each protein–microbe pair (p, q), we created the data set {(*l*_*pi*_, *a*_*qj*_ *): s*_*i*_ *= s*_*j*_} generated from all protein experiments *i* and microbe experiments *j*, where *l*_*pi*_ is the measured level of *p* in the experiment *i*, *a*_*qj*_ is the measured abundance of *q* in experiment *j*, and *s*_*i*_ and *s*_*j*_ are the substrates used in experiments *i* and *j*. This procedure presumes that all collinearity between protein levels and microbe abundance values is due to substrates, i.e., protein levels and microbe abundance values are independent across samples applied to the same substrate.

First, we calculated two measures of the strength of the relationship between each protein *p* and microbe *q*: the classical Pearson correlation coefficient, which measures the linear relationship between the two variables, and mutual information, an information-theoretic measure of the dependence of each variable on the other. Canonical correlation analysis (CCA) was then used to identify the strongest associations among linear combinations (weighted sums) of the respective species. Only those proteins and microbes that discriminated among the substrates in the LDA were included. The interset correlations of the predictors and the intraset correlations of the responses were then superimposed in a biplot. In this plot, predictor and response vectors are more closely aligned (or opposing) when the correlation is strong and positive (or negative), and longer intraset vectors indicate microbes whose abundance values are more accurately predicted. This approach was used to correlate protein levels with microbe abundance values at both 2 and 24 h and to correlate microbe abundance values at 2 h with those at 24 h.

#### Software

The working environment considered: Tidyverse package collection,^86^ the ‘candisc’ package for CCA, the ‘broom’ package from the Tidymodels collection,^87^ and the ‘ordr’ package that extends Tidyverse principles to ordination methods.^88^

## Supplementary Material



## Data Availability

Sequence data for metagenome have been deposited in the Sequence Read Archive (SRA) data from National Center for Biotechnology information (NCBI) under accession number PRJNA1104242 (https://dataview.ncbi.nlm.nih.gov/object/PRJNA1104242?reviewer=eidvn37t875m72kkcmnpcl96). Proteomic data is available in the Harvard Dataverse (10.7910/DVN/FSFMFT).

## Notes

Supplemental results (PDF)
