# Supplementary material for: Exploring the Impact of Biotic and Abiotic Surfaces on Protein Binding Modulation and Bacteria Attachment: Integrating Biological and Mathematical Approaches
Source: ACS Nano. 2025 Jun 16;19(25):23393–413. doi: 10.1021/acsnano.5c06573 (PMC12224314; doi:10.1021/acsnano.5c06573)
Supplement: Supplementary file 1 [file nn5c06573_si_001.pdf]

Supplemental results

Exploring the impact of biotic and abiotic surfaces on protein binding modulation and bacteria attachment: integrating biological and mathematical approaches

João Gabriel S. Souza, Martinna Bertolini, Jett Liu, Bruna Egumi Nagay, Rodrigo Martins, Raphael C. Costa, Jason Cory Brunson, Jamil Shibli, Luciene Cristina Figueiredo, Anna Dongari-Bagtzoglou, Magda Feres, Valentim Adelino Ricardo Barã[#], Batbileg Bor

Table S1. Exclusive proteins identified in each surface exposed to saliva

ID protein	Protein name	Molecular function
Titanium		
K7ER74	Apolipoprotein C-II	enzyme activator activity
A0A024RA52	Proteasome subunit alpha type	threonine-type endopeptidase activity
P08670	Vimentin	structural constituent of cytoskeleton
P09525	Annexin A4	calcium ion binding
P08758	Annexin A5	phospholipid binding
F8W1U5	DUF3456 domain-containing protein	
P04839	Cytochrome b-245 heavy chain	metal ion binding
P06310	Immunoglobulin kappa variable 2-30	antigen binding
P13796	Plastin-2	calcium ion binding
P31146	Coronin-1A	cytoskeletal protein binding
P41218	Myeloid cell nuclear differentiation antigen	double-stranded DNA binding
Q9C0B6	BMP/retinoic acid-inducible neural-specific protein 2	cell cycle arrest
Enamel		
P04259	Keratin, type II cytoskeletal 6B	structural constituent of cytoskeleton
Q96AX9-14	E3 ubiquitin-protein ligase MIB2	zinc ion binding
P16401	Histone H1.5	nucleosomal DNA binding
Dentin		
Q14533	Keratin, type II cuticular Hb1	keratinization
A0A096LPE2	SAA2-SAA4 readthrough	acute-phase response
P45880	Voltage-dependent anion-selective channel protein 2	nucleotide binding
A0A0C4DG17	40S ribosomal protein SA	structural constituent of ribosome
Q9Y6R0	Numb-like protein	regulation of neurogenesis
A0A140T8X8	Mucin-21	
P09972	Fructose-bisphosphate aldolase C	ructose-bisphosphate aldolase activity
P43251-2	Biotinidase	biotinidase activity
P02663	Alpha-S2-casein	zymogen binding
Q6KB66-3	Keratin, type II cytoskeletal 80	
Q15323	Keratin, type I cuticular Ha1	structural constituent of cytoskeleton
D6REE5	Receptor of-activated protein C kinase 1	
P05120	Plasminogen activator inhibitor 2	serine-type endopeptidase inhibitor activity
P02511	Alpha-crystallin B chain	metal ion binding
F5H5D3	Tubulin alpha chain	GTP binding
P01033	Metalloproteinase inhibitor 1	zinc ion binding
H7BZJ3	Protein disulfide-isomerase A3	protein disulfide isomerase activity
P61019	Ras-related protein Rab-2A	GTP binding
M0R0R2	40S ribosomal protein S5	RNA binding

O14791-2	Apolipoprotein L1	lipid binding
O15231-6	Zinc finger protein 185	zinc ion binding
O60235	Transmembrane protease serine 11D	peptidase activity
P02748	Complement component C9	
P07476	Involucrin	
P10643	Complement component C7	complement activation
P11234-2	Ras-related protein Ral-B	GTP binding
P13489	Ribonuclease inhibitor	ribonuclease inhibitor activity
P14174	Macrophage migration inhibitory factor	cytokine activity
P22735	Protein-glutamine gamma-glutamyltransferase K	metal ion binding
P22792	Carboxypeptidase N subunit 2	enzyme regulator activity
P31946	14-3-3 protein beta/alpha	phosphoprotein binding
P62258	14-3-3 protein epsilon	enzyme binding
P68431	Histone H3.1	DNA binding
Q562R1	Beta-actin-like protein 2	ATP binding
Q6XPR3	Repetin	calcium ion binding
Q86T26	Transmembrane protease serine 11B	serine-type peptidase activity
Q92597	Protein NDRG1	cadherin binding
Q96FQ6	Protein S100-A16	calcium ion binding
Q9BW60	Elongation of very long chain fatty acids protein 1	fatty acid elongase activity
Q9BYD5	Cornifelin	
Q9BZQ8	NIBAN1	
Q9Y446-2	Plakophilin-3	cell adhesion molecule binding

Figure S1. Clustered heatmap of shared proteins among the artificial (titanium) and natural (dentine and enamel) surfaces with a statistical difference ($p<0.05$, Kruskal-Walis test) in terms of LFQ intensity.

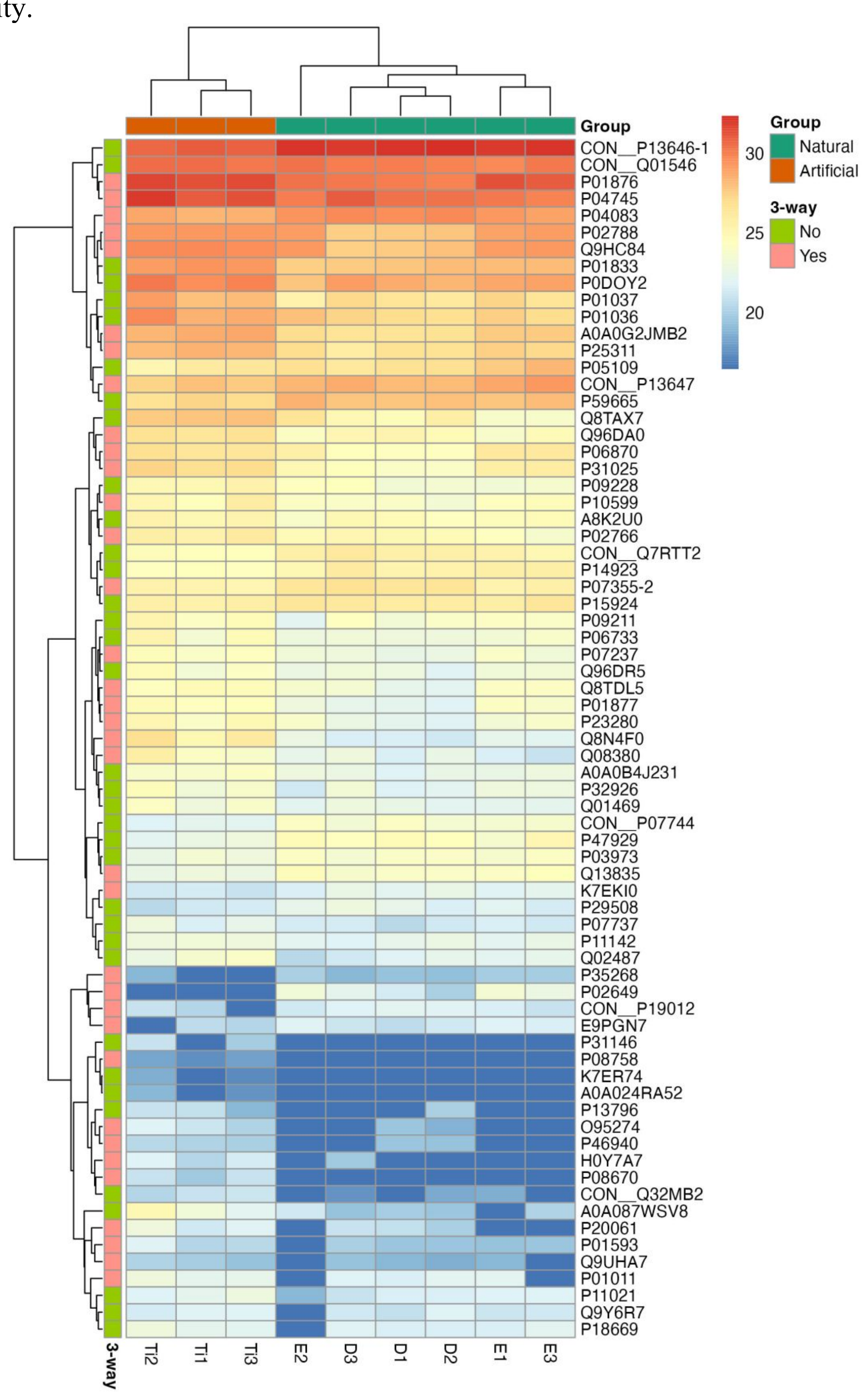

Figure S2. LFQ intensity of all proteins identified on salivary pellicle formed (2 h) on titanium (T), enamel (E) and dentine (D).

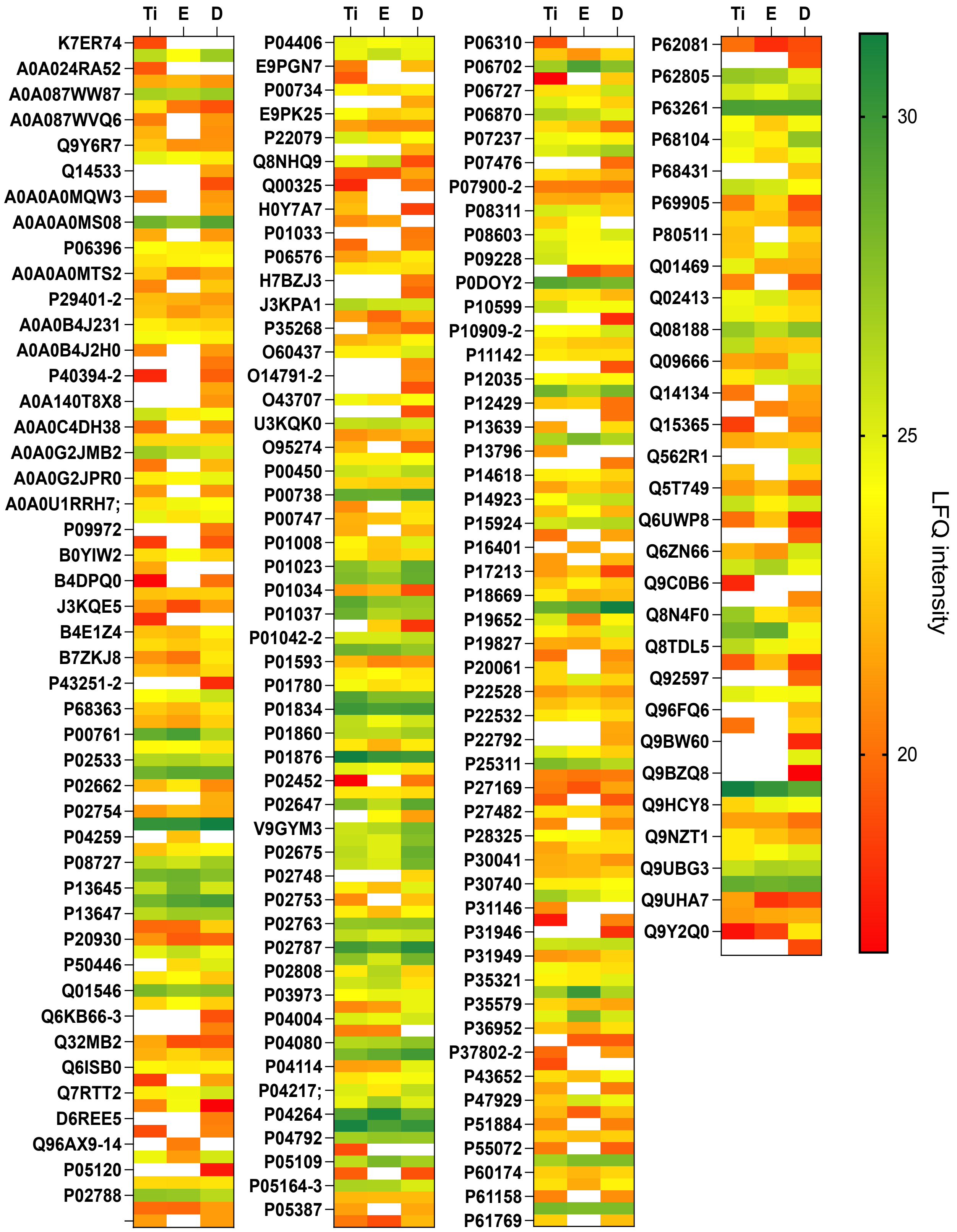

Figure S3. Log-offset-abundances in RNA-sequencing experiments of microbes at 2 hours that differ by substrate using Kruskal-Wallis test ($p<0.05$).

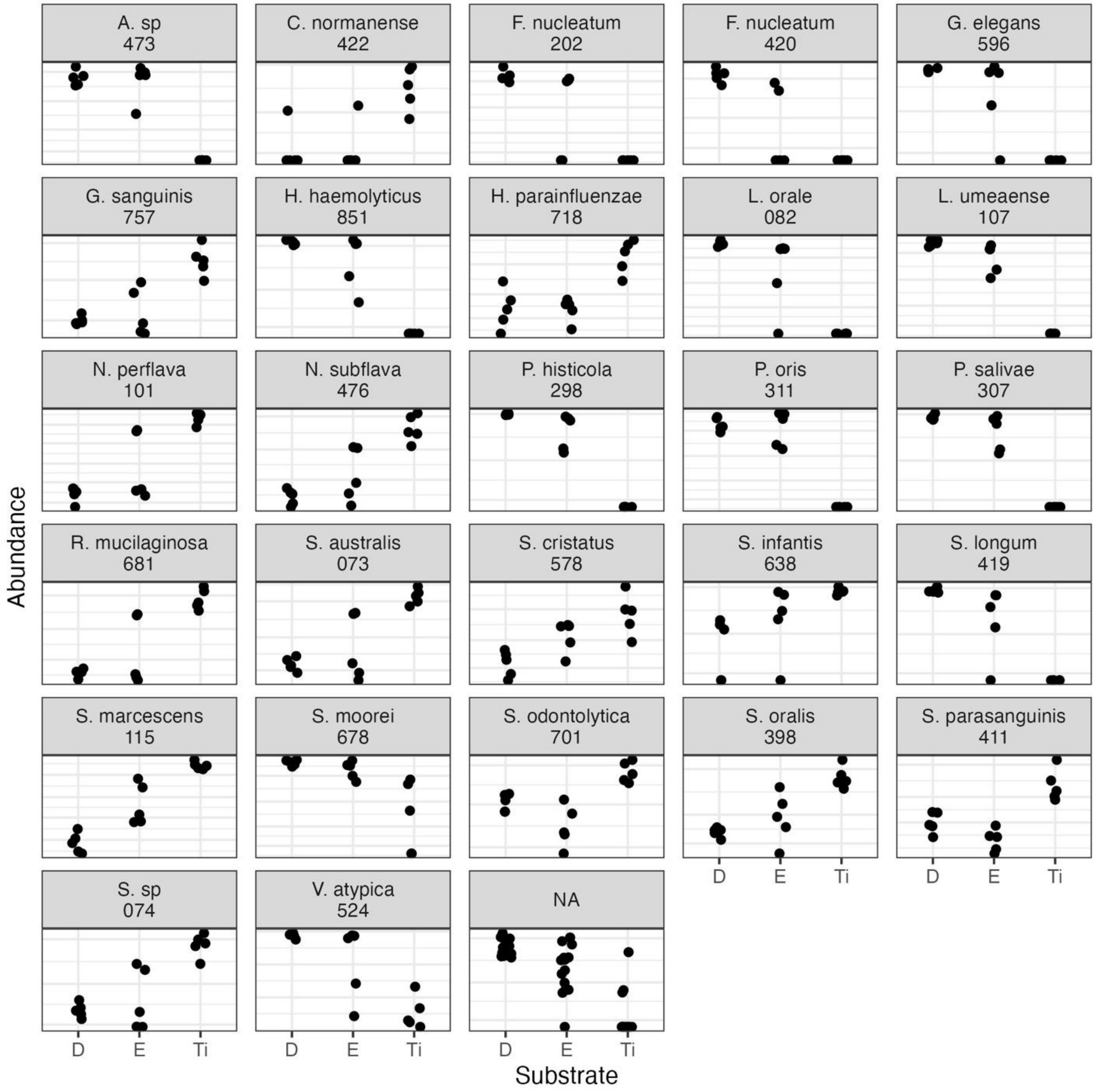

Figure S4. Methicillin-resistant Staphylococcus aureus (MRSA) adhesion was evaluated over a 2-hour period on enamel (E), dentine (D), and titanium (Ti) surfaces, both in the absence and presence of a salivary pellicle (SP). For the SP groups, the pellicle was formed by incubating the substrates with stimulated human saliva for 2 hours prior to bacterial exposure. Bacterial adhesion was quantified as colony-forming units (CFU)/cm2. Statistically significant differences are indicated by asterisks: **p < 0.01, ***p < 0.001, and ****p<0.0001.

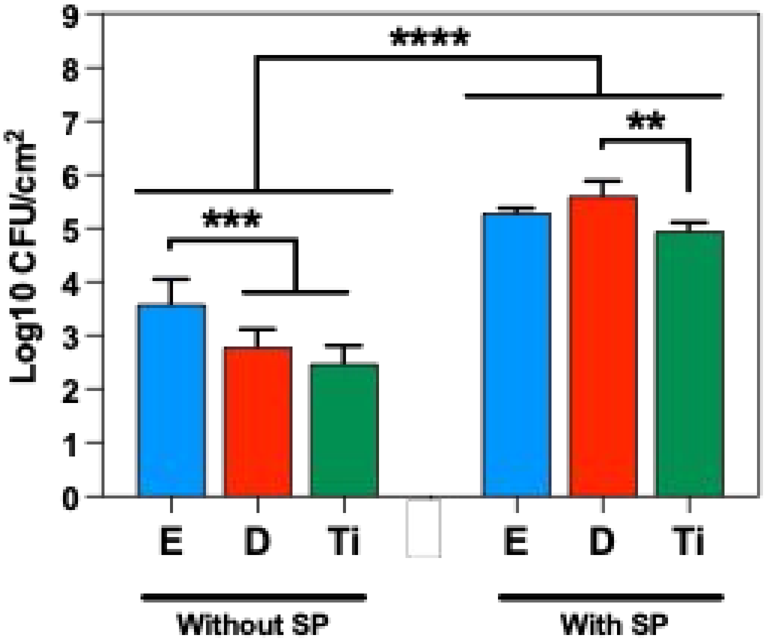

Figure S5. Microbiome analysis of 2 and 24 hours after microbial adhesion and initial biofilm formation. (A) Principal coordinates analysis (PCoA) using the Bray-Curtis distance function and ASV abundances. Microbial adhesion (2 hours) and accumulation (24 hours) was conducted using stimulated human saliva as microbial inoculum. The microbiome profile was evaluated by 16S RNA sequencing. (B) Alpha diversity analysis by Shannon Index of sequenced samples from each substrate. (C) Stacking bar charts showing the relative abundance of species identified within sequenced samples.

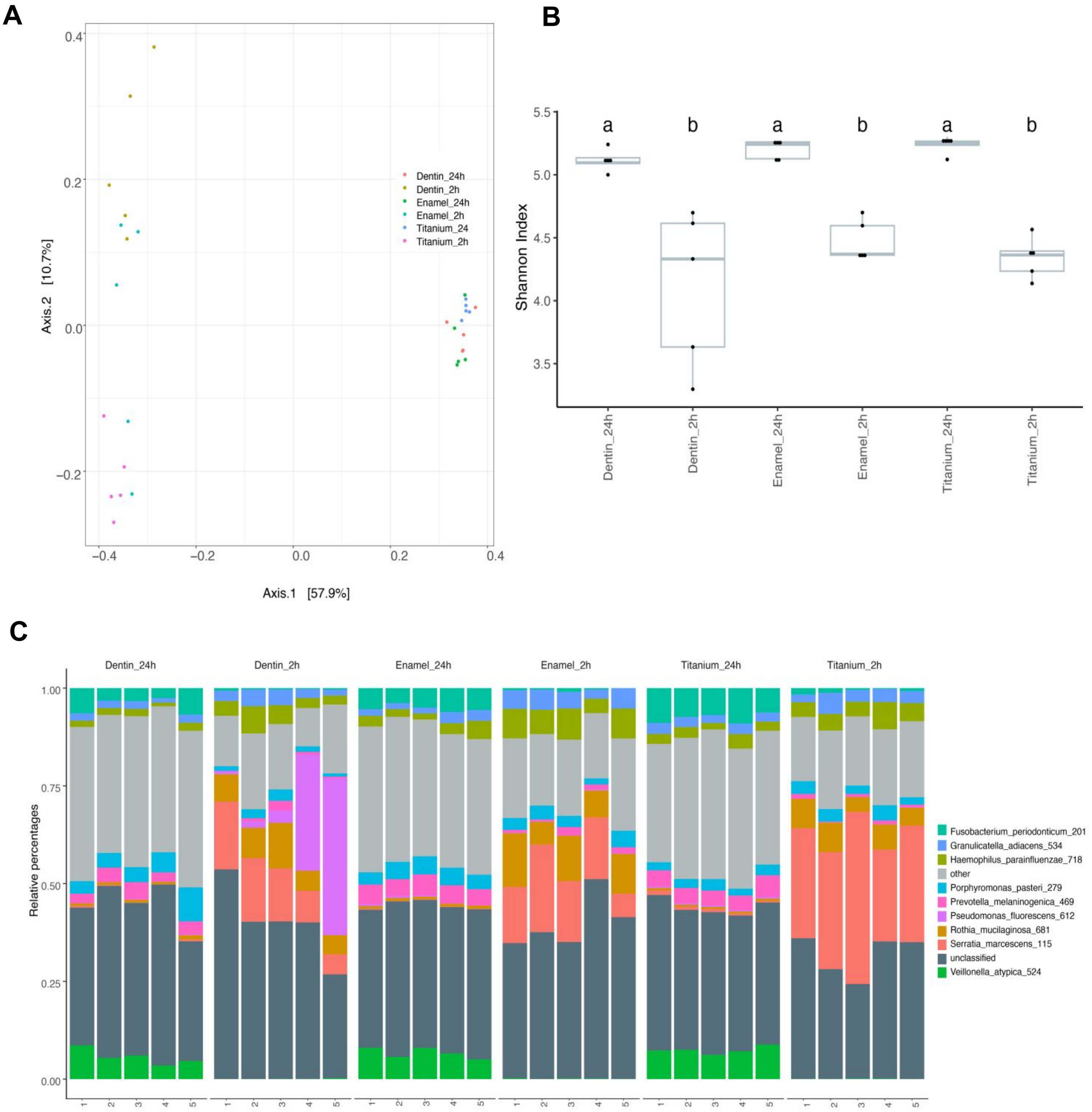

Figure S6. Canonical correlation analysis (CCA) to correlate protein and microbial species. (A) Pearson correlations between matched-substrate bootstrapped samples of protein levels and microbe abundances at 2 hours.

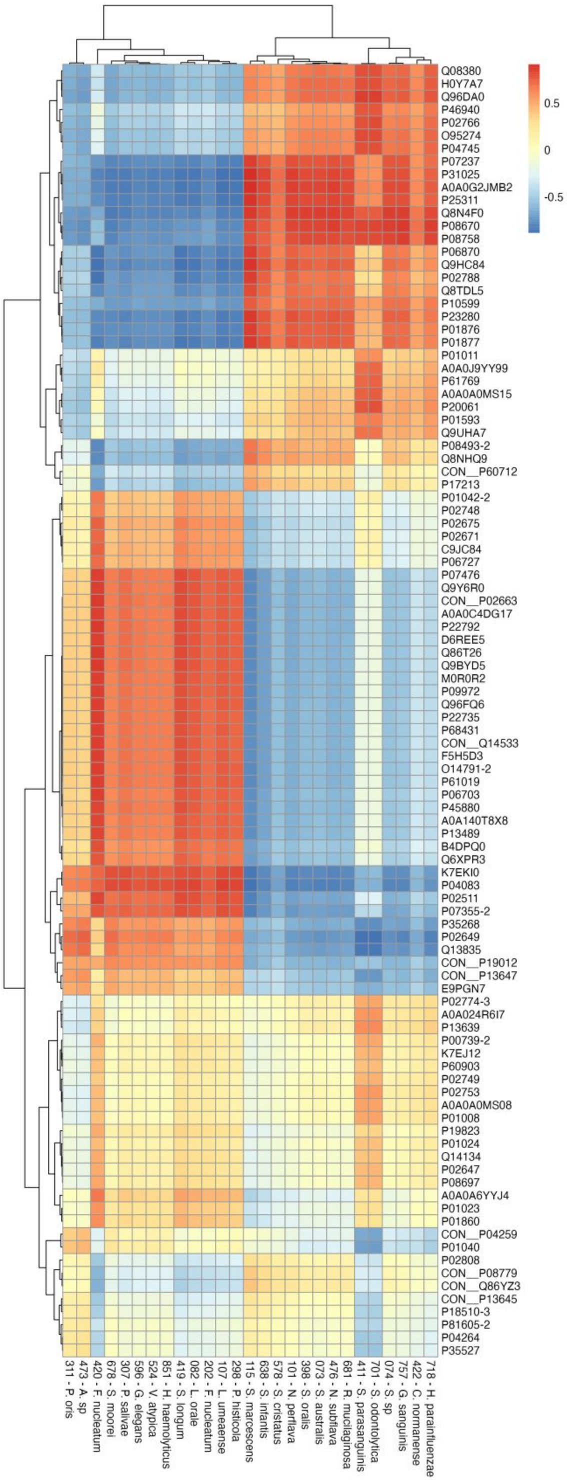

Table S2. Canonical correlation analysis (CCA) to correlate protein and microbial species. CCA analysis of proteomic data and microbial adhesion (2 hours) showing only proteins highly correlated (>0.7) to microbes

Microbe HMT number	Protein code	Correlation in 2CDs		Microbe	Protein	Correlation in 2CDs
**74**	P04083	-799		**420**	B4DPQ0	791
**74**	K7EKI0	-772		**420**	P13489	827
**74**	P02649	-772		**420**	P06703	842
**74**	Q13835	-733		**420**	P61019	846
**74**	P01876	710		**420**	P07476	855
**74**	H0Y7A7	727		**420**	Q9BYD5	858
**74**	Q96DA0	736		**578**	A0A0G2JMB2	706
**74**	P07237	769		**596**	A0A0G2JMB2	-803
**74**	P25311	797		**596**	P25311	-791
**74**	A0A0G2JMB2	813		**596**	P07237	-760
**74**	P08758	829		**596**	Q9HC84	-760
**82**	Q9HC84	-847		**596**	P01876	-741
**82**	A0A0G2JMB2	-845		**596**	P08758	-737
**82**	P25311	-835		**596**	P06870	-732
**82**	P06870	-823		**596**	P02788	-713
**82**	P02788	-815		**596**	P07355-2	712
**82**	P01876	-806		**596**	K7EKI0	760
**82**	P07237	-800		**596**	P04083	785
**82**	P08758	-720		**678**	A0A0G2JMB2	-838
**82**	P10599	-711		**678**	P25311	-825
**82**	Q6XPR3	702		**678**	P08758	-801
**82**	P13489	747		**678**	P07237	-793
**82**	P06703	751		**678**	Q9HC84	-766
**82**	P61019	764		**678**	P01876	-758
**82**	P07476	772		**678**	P06870	-734
**82**	Q9BYD5	774		**678**	P10599	-711
**82**	P07355-2	781		**678**	P02788	-708
**82**	K7EKI0	798		**678**	P02649	711
**82**	P04083	823		**678**	P07355-2	726
**202**	Q9HC84	-790		**678**	K7EKI0	795
**202**	A0A0G2JMB2	-786		**678**	P04083	821
**202**	P25311	-777		**681**	P04083	-804
**202**	P06870	-768		**681**	K7EKI0	-778
**202**	P02788	-761		**681**	P02649	-716
**202**	P01876	-751		**681**	P07355-2	-703
**202**	P07237	-744		**681**	P06870	706
**202**	P06703	702		**681**	P01876	736
**202**	P61019	714		**681**	Q9HC84	738
**202**	P07476	721		**681**	P07237	776
**202**	Q9BYD5	724		**681**	P08758	797
**202**	P07355-2	728		**681**	P25311	806
**202**	K7EKI0	742		**681**	A0A0G2JMB2	820
**202**	P04083	765		**701**	P02649	-876
**411**	P02649	-876		**701**	Q13835	-853
**411**	Q13835	-852		**701**	CON__P13647	-734
**411**	CON__P13647	-730		**701**	P04745	797
**411**	P04745	795		**701**	H0Y7A7	800
**411**	H0Y7A7	801		**701**	P02766	806
**411**	P02766	803		**701**	P08758	812
**411**	P08758	817		**701**	Q96DA0	857
**411**	Q96DA0	856		**718**	P02649	-814
**420**	P02788	-868		**718**	Q13835	-781
**420**	Q9HC84	-866		**718**	P04083	-743
**420**	P06870	-853		**718**	K7EKI0	-716
**420**	P01876	-790		**718**	P02766	700
**420**	P08493-2	-789		**718**	P07237	710
**420**	A0A0G2JMB2	-776		**718**	P04745	716
**420**	P25311	-771		**718**	P25311	734
**420**	P07237	-734		**718**	A0A0G2JMB2	751
**420**	P02671	722		**718**	H0Y7A7	758
**420**	K7EKI0	728		**718**	Q96DA0	784
**420**	P04083	749		**718**	P08758	829
**420**	P07355-2	774				
**420**	Q6XPR3	778				

Table S3. Canonical correlation analysis (CCA) to correlate protein and microbial species. CCA analysis of proteomic data and microbial accumulation (24 hours) showing only proteins highly correlated (>0.7) to microbes.

**Microbe HMT number**	**Protein code**	**Correlation in 2CDs**		**Microbe**	**Protein**	**Correlation in 2CDs**
**213**	P04083	-828		**763**	P25311	742
**213**	K7EKI0	-799		**763**	A0A0G2JMB2	752
**213**	Q13835	-787		**780**	P04083	-847
**213**	Q9HC84	707		**780**	K7EKI0	-819
**213**	P04745	715		**780**	Q13835	-751
**213**	P10599	718		**780**	P07355-2	-726
**213**	P01876	726		**780**	P06870	720
**213**	H0Y7A7	776		**780**	P10599	735
**213**	P07237	795		**780**	H0Y7A7	749
**213**	P25311	824		**780**	Q9HC84	756
**213**	A0A0G2JMB2	840		**780**	P01876	762
**213**	P08758	875		**780**	P07237	816
**311**	P04083	-814		**780**	P25311	847
**311**	K7EKI0	-786		**780**	A0A0G2JMB2	863
**311**	Q13835	-730		**780**	P08758	863
**311**	P10599	705		**917**	P04083	-775
**311**	Q9HC84	720		**917**	K7EKI0	-750
**311**	H0Y7A7	727		**917**	P06870	701
**311**	P01876	728		**917**	P01876	720
**311**	P07237	783		**917**	Q9HC84	731
**311**	P25311	813		**917**	P08758	748
**311**	A0A0G2JMB2	828		**917**	P07237	749
**311**	P08758	834		**917**	P25311	779
**524**	P04083	-791		**917**	A0A0G2JMB2	792
**524**	K7EKI0	-765		**930**	P04083	-785
**524**	P07355-2	-702		**930**	K7EKI0	-760
**524**	P06870	712		**930**	P07355-2	-712
**524**	P01876	733		**930**	P02788	712
**524**	Q9HC84	742		**930**	P06870	731
**524**	P07237	764		**930**	P08758	738
**524**	P08758	766		**930**	P01876	740
**524**	P25311	794		**930**	Q9HC84	759
**524**	A0A0G2JMB2	807		**930**	P07237	760
**686**	P02788	-714		**930**	P25311	791
**686**	P61019	713		**930**	A0A0G2JMB2	803
**686**	P07476	720		**945**	P04083	-760
**686**	Q9BYD5	723		**945**	K7EKI0	-736
**763**	P04083	-734		**945**	P01876	705
**763**	K7EKI0	-711		**945**	Q9HC84	714
**763**	P06870	701		**945**	P07237	734
**763**	P01876	702		**945**	P08758	736
**763**	P07237	712		**945**	P25311	764
**763**	Q9HC84	725		**945**	A0A0G2JMB2	776

Table S4. Canonical correlation analysis (CCA) to correlate microbial species. CCA analysis of microbial adhesion (2 hours) and microbial accumulation (24 hours) showing species highly correlated (>0.5).

**Microbe HMT number (24 h)**	**Microbe HMT number (2 h)**	**Correlation in 2CDs**
**213_24h**	420_2h	-597
**213_24h**	701_2h	735
**311_24h**	420_2h	-612
**311_24h**	701_2h	682
**524_24h**	420_2h	-637
**524_24h**	701_2h	593
**686_24h**	420_2h	623
**763_24h**	420_2h	-628
**780_24h**	420_2h	-643
**780_24h**	701_2h	701
**917_24h**	420_2h	-628
**917_24h**	701_2h	576
**930_24h**	420_2h	-655
**930_24h**	701_2h	553
**945_24h**	420_2h	-614
**945_24h**	701_2h	568
